# Regulating the lncRNA DSCR9/RPLP2/PI3K/AKT axis: an important mechanism of Xinfeng capsules in improving rheumatoid arthritis

**DOI:** 10.3389/fimmu.2024.1465442

**Published:** 2024-09-23

**Authors:** Fanfan Wang, Jian Liu

**Affiliations:** ^1^ The First Affiliated Hospital of Anhui University of Chinese Medicine, First Clinical Medical College, Hefei, Anhui, China; ^2^ Department of Rheumatism Immunity, The First Affiliated Hospital of Anhui University of Chinese Medicine, Hefei, Anhui, China

**Keywords:** Xinfeng capsules, rheumatoid arthritis, lncRNA DSCR9, inflammation, hypercoagulability

## Abstract

**Background:**

Rheumatoid arthritis (RA) is a systemic autoimmune disease characterized by chronic and symmetrical polyarthritis. RA patients often experience inflammatory reaction and hypercoagulable state, which together affect the self-perception of patient (SPP). Currently, inhibiting inflammation and hypercoagulable state are common treatment methods for alleviating RA symptoms. Xinfeng Capsules (XFC) has a long history of treating RA, and can effectively improve the inflammatory response and hypercoagulable state of RA. However, the potential mechanisms have not yet been determined.

**Purpose and study design:**

This study elucidated the action mechanism of XFC in RA inflammation and hypercoagulability through the lncDSCR9/RPLP2/PI3K/AKT axis.

**Results:**

Clinical observations indicated that there was a strong link between XFC therapy and improvements in inflammatory and coagulation biomarkers, as well as SPP among RA patients. The subsequent network pharmacology analysis results identified the PI3K/AKT signaling pathway as a potential mediator for XFC treatment of RA. Furthermore, clinical validation and sequencing results revealed that lncRNA DSCR9 expression (a gene implicated in inflammation and coagulation) was negatively correlated with clinical markers of inflammation and coagulation, while positively correlated with SF-36 indicators. Notably, XFC treatment remarkably upregulated lncRNA DSCR9 expression and downregulated PI3K and AKT expressions, showing opposite expression trends to the untreated cases.The regulatory effect of XFC on the lncRNA DSCR9/RPLP2/PI3K/AKT axis in RA was investigated using techniques such as RNA pull-down assay, Western blot analysis, RT-PCR, and EdU assay. Moreover, the administration of the PI3K/AKT agonist RMH can counteract the effects of XFC on p-PI3K, p-AKT, inflammation, and hypercoagulability, reinforcing the role of pathway. Finally, animal studies utilizing HE staining and transmission electron microscopy (TEM) demonstrated that XFC notably decreased PI3K and AKT expressions in adjuvant-induced arthritis (AA) rats, mitigated inflammation and hypercoagulability, and enhanced the ultrastructure of synovial cells. These findings underscored the potential mechanisms of XFC in the treatment of RA.

**Conclusion:**

Regulating the lncRNA DSCR9/RPLP2/PI3K/AKT axis may be an important mechanism by which XFC improved RA inflammatory response and hypercoagulable state.

## 1 Introduction

Rheumatoid arthritis (RA) is a chronic symmetric inflammatory autoimmune disease characterized by polyarthritis, encompassing joint inflammation, synovial hyperplasia, and destruction of cartilage and bone (1). RA has a global incidence rate of 0.5-1.0% and predominantly affects women (2).The underlying mechanisms of RA are associated with genetics, immunity, and infection, but they are not yet clear (3, 4). The treatment strategies aim to detect and control the progression early, and the traditional therapies include non-steroidal anti-inflammatory drugs (NSAIDs), glucocorticoids (GCs), and disease-modifying anti-rheumatic drugs (DMARDs), which alleviate inflammation, suppress immunity, and prevent disease progression, respectively (5). Nevertheless, approximately 30% of patients have poor therapeutic outcomes, accompanied by severe side effects (such as gastrointestinal reactions, liver and kidney damage, and cardiovascular issues), which significantly affect patients’ quality of life (6–9). Therefore, it is of great necessity to develop safer and more effective therapeutic agents for RA treatment.

Immunological inflammation plays a pivotal role in RA, and persistent inflammatory stimuli can lead to joint swelling, pain, deformity, functional decline, and eventually reduction in patients’ quality of life (10). The synovial tissue serves as the epicenter of RA inflammation, with fibroblast-like synoviocytes (FLSs) as key players that facilitate synovial inflammation and cartilage-bone damage (11, 12). The activated FLSs release a large amount of inflammatory factors and exhibit tumor-like cellular behaviors, exacerbating disease progression (13). It has been shown that a characteristic pathological product is formed during RA pathogenesis: the pannus composed of neo-microvasculature, proliferative and hypertrophic FLSs, and inflammatory cells. This tumor-like pannus is the main cause and pathological basis of joint lesions and cartilage destruction (14). Angiogenesis is a vital process in RA progression, with intravascular coagulation and microcirculatory thrombosis being the most common pathophysiological changes during its progressive development (15). Additionally, our previous clinical trials have observed a large number of clinical trial indicators and consistently revealed frequently abnormal coagulation and fibrinolysis indicators in RA patients, indicative of a hypercoagulable state (16, 17). Therefore, modulating inflammation and coagulation represents a crucial therapeutic direction for RA management.

Long non-coding RNAs (lncRNAs) can regulate gene expression through various mechanisms, including chromatin modification, mRNA processing, and other intricate pathways. Notably, lncRNAs display dysregulated expression patterns in inflammatory disorders such as RA (18–20). In our research, peripheral blood mononuclear cells (PBMCs) from early-stage RA patients were utilized and evaluated using high-throughput transcriptome sequencing to analyze the expression profile of RA-associated lncRNAs. Among these, lncRNA DSCR9 (lncDSCR9 or DSCR9) was identified, which is a differentially expressed lncRNA implicated in inflammation and hypercoagulability (21). Multiple studies have shown that lncDSCR9 is abnormally expressed in various diseases, which can inhibit cell proliferation and promote cell apoptosis (22, 23). However, the specific mechanisms of lncDSCR9 in RA remain to be elucidated. Thus, elucidating these aspects holds significant promise for advancing RA therapeutics. It has been shown that the phosphatidylinositol-3-kinase (PI3K)/protein kinase B (AKT) signaling pathway is abnormally activated in RA synovial cells, regulating inflammation, cell proliferation, and cell apoptosis, and contributing to RA pathology (24). Inhibition of the PI3K/AKT pathway can induce FLS apoptosis, showing therapeutic potential for RA (25). Furthermore, PI3K/AKT activates interleukin (IL)-1β to induce the expression of tumor necrosis factor-alpha (TNF-α) and IL-6, thereby exacerbating inflammation (26). Additionally, the PI3K/AKT pathway can promote endothelial cell proliferation and migration, thus participating in angiogenesis and vascular remodeling, which are intimately linked to the hypercoagulable state of RA patients (27). Thus, modulation of this signaling network holds promise for addressing multiple facets of RA pathology.

The adjuvant-induced arthritis (AA) rat model is an inflammatory model widely used to explore the mechanisms of RA, exhibiting clinical and pathological hallmarks similar to human RA (28). In the realm of traditional Chinese medicine (TCM), Tripterygium wilfordii and its derivatives are commonly used for managing immune-mediated disorders, such as RA and systemic lupus erythematosus (29). Triptolide (TPL) is the main bioactive constituent of Tripterygium wilfordii, which has potent anti-inflammatory and immunosuppressive properties and therefore has been applied in the treatment and research of RA (30, 31).

Xinfeng Capsule (XFC, Anhui Pharmaceutical Production Number: Z20050062; Patent Number: ZL 2013 1 0011369.8) is a formulation exclusively developed by the First Affiliated Hospital of Anhui University of Chinese Medicine. XFC formula includes Astragalus membranaceus, Coicis semen, Tripterygium wilfordii, and centipede, with a strict ratio of 20:20:10:1. A previous study has confirmed that XFC has rigorous quality assurance, which has been evidenced by high-performance liquid chromatography (HPLC) fingerprinting (32). XFC has been clinically applied for many years and has demonstrated significant advantages in the treatment of RA (33). Research has indicated that XFC can inhibit RA-specific FLS (RA-FLS) proliferation, regulate inflammatory factors and oxidative stress, reduce IL-6 and IL-17 levels, and enhance Nrf2, HO-1 expressions (34). Clinic data have suggested that XFC may improve RA-associated inflammation and hypercoagulability, potentially through mechanisms related to regulating immune-inflammatory and coagulation indicators (16). Furthermore, it has been shown that XFC can alleviate RA symptoms by inhibiting abnormal activation of the PI3K pathway and reducing IL-1β, IL-33, C-C motif chemokine ligand 5 (CCL5), and vascular endothelial growth factor (VEGF) expressions (35). Despite the revealed biological effects of XFC, its specific mechanisms on RA hypercoagulability and inflammatory responses have not been validated.

In this study, we investigated the effects of XFC on clinical inflammatory indicators, coagulation indicators, and self-perception of patient (SPP) in RA patients. Clinical samples of RA patients were collected for validation. Additionally, *in vivo* and *in vitro* RA models were constructed to investigate the role and mechanism of the lncRNA DSCR9/RPLP2/PI3K/AKT axis in the therapeutic effect of XFC on RA inflammation and hypercoagulability state.

## 2 Materials and methods

### 2.1 Clinical data retrieval

The comprehensive clinical data of 326 discharged RA patients were collected from the electronic health recordsystem of the Rheumatology Department of the esteemed First Affiliated Hospital of Anhui University of Chinese Medicine. Amongst these cases, 151 patients underwent XFC treatment.A broad spectrum of indicators were encompassed in this research, includingclinical indicators [platelet count (PLT), fibrinogen (FBG), D-dimer (DD), erythrocyte sedimentation rate (ESR), high-sensitivity C-reactive protein (Hs-CRP), rheumatoid factor (RF), and anti-cyclic citrullinated peptide antibodies (CCP)]and SPP indicators [self-rating anxiety scale (SAS) score, self-rating depression scale (SDS) score, visual analog scale (VAS) score, and the medical outcomes study (MOS) 36-item short-form health survey (SF-36) assessing 8 domains: physical functioning (PF), role physical (RP), body pain (BP), general health (GH), vitality (VT), social functioning (SF), role emotional (RE), and mental health (MH)].

### 2.2 Association rules

XFC treatment was set as “T” and unused as “F”, with indicators improved as “T” and indicators not improved as “F” after treatment. The association rule analysis between the use of XFC and the improvement of indicators was conducted using the Apriori module in IBM SPSS Modeler 18.0 software, with the support, confidence, and improvement calculated following the calculation formulas in previous research (36).

### 2.3 Network pharmacology

With the pharmacokinetic criteria of oral bioavailability(OB) ≥ 30% and drug-like (DL) ≥ 0.18, the active compounds and their corresponding targets of 4 TCMs were screened through the TCMSP database. Subsequently, RA-related genes were retrieved from GeneCards, OMIM, DrugBank, and TTD databases. Next, the overlapping genes between XFC active compounds and the RA targets were identified using RData software. Following this, KEGG pathway analysis was conducted on these overlapping genes to gain insights into their biological functions and interactions.

### 2.4 Collection and separation of PBMCs in clinical samples

A total of 50 hospitalized RA patients from the Rheumatology Department at the First Affiliated Hospital of Anhui University of Traditional Chinese Medicine were enrolled. Peripheral blood samples were obtained before and after XFC treatment and RA-specific PBMCs (RA-PBMCs) were isolated utilizing a density gradient method (Histopaque-1077, Sigma). All participants met the diagnostic criteria for RA, established by the American College of Rheumatology (ACR) and the European League Against Rheumatology (EULAR) in 2010 (37). Meanwhile, a total of 30 healthy volunteers, matched for age and gender, were recruited from the Physical Examination Center of the same hospital. This research strictly adhered to the ethical principles of the Helsinki Declaration. During the follow-up process, we ensured comprehensive privacy protection for patients and no disruption to their treatment plans. This study was approved by the Ethics Committee of the First Affiliated Hospital of Anhui University of Traditional Chinese Medicine, and all participants signed the informed consent (Ethics Number: 2019AH-12).

### 2.5 Cell co-culture

RA-FLSs were obtained from Beijing Beina Chuanglian Biotechnology Research Institute and identified through short tandem repeat (STR) profiling. RA-FLSs were isolated and cultured following established protocols outlined by Wen et al. (31), with excess cells discarded. The remaining RA-FLSs were digested with 0.25% trypsin and resuspended in a complete Dulbecco’s Modified Eagle Medium (DMEM). To establish a co-culture system, RA-FLSs and RA-PBMCs (both 5 x10^5^ cells/well) were seeded into Transwell chambers and maintained in a cell culture incubator (37°C, 5% CO_2_).

### 2.6 Cell transfection

For transfection, the co-cultured RA-FLSs were collected and seeded in DMEM medium supplemented with 10% fetal bovine serum (FBS) in 24-well plates. The cells were incubated overnight in a cell culture incubator, (37°C, 5% CO_2_). Subsequently, 5μL of the transfection reagent (Lipofectamine 2000) was introduced to the cells. The overexpression plasmids/siRNA wasmixed with DMEM medium, followed by incubation (48 h, 37°C) with the cells. Cells were collected and subjected to reverse transcription, and gene expression levels were quantified using reverse transcription-quantitative polymerase chain reaction (RT-qPCR) to assess the transfection efficiency.

### 2.7 RT-qPCR analysis

Cells and supernatants were collected from 50 RA patients and 30 healthy volunteers with PrimeScript™RT reagent Kit with gDNA Eraser kit following the manufacturer’s instructions. Cell RNA was extracted using the TRIzol method for the amplification reaction. PCR products were analyzed through agarose gel electrophoresis, and relative quantitative analysis was performed using the 2^-△△Ct^ method, with β-actin expression as a reference. The primer sequences for each detection indicator are shown in **Table 1**.

**Table 1:** PCR real-time primer sequences.

Gene	Amplicon Size (bp)	Forward primer (5’→3’)	Reverse primer (5’→3’)
Hu-β-actin	96	CCCTGGAGAAGAGCTACGAG	GGAAGGAAGGCTGGAAGAGT
Hu-Lnc-DSCR9	133	AGAGGTAACAGAGCCCTTCG	CACCTCGCTTCATTTGGGTT
Hu-RPLP2	93	CCGGCTCAACAAGGTTATCA	CCAGCAGGTACACTGGCAA
Hu-PI3K	138	TGTGGAGCTCGCTAAAGTCA	CACTCCTGCCCTAAATGGGA
Hu-AKT	167	CTTTCGGCAAGGTGATCCTG	GTACTTCAGGGCTGTGAGGA

### 2.8 Western blot analysis

Total protein was extracted from co-cultured RA-FLSs or PBMCs using a radioimmunoprecipitation assay (RIPA) lysis buffer.After protein quantification, samples were then loaded onto a gel for electrophoresis, followed bymembrane transfer. The membranes were blocked (room temperature, 2 h) in Western blot blocking solution [containing 5% skimmed milk powder or 5% bovine serum albumin (BSA) for phosphorylated proteins], and then incubated with primary antibodies against PI3K (1:500), p-PI3K (1:500), AKT (1:500), p-AKT (1:500), and RPLP2 (1:1000). After washing, a horseradish peroxidase (HRP)-conjugated secondary antibody (1:10000) was added for incubation (room temperature, 2 h), followed by 3 washeswith PBST detergent (10 min each). After that, the protein was detected using an enhanced chemiluminescence assay kit. Finally, the protein blot data were quantified and analyzed using the ImageJ software.

### 2.9 Enzyme-linked immunosorbent assay

The supernatant of the co-cultured RA-FLSs was collected and centrifuged (1000r/min, 10 min). Next, 100 μL of supernatant was added to each well of the ELISA plate for incubation (37°C, 1.5 h). The concentration of cytokines [IL-6, IL-8, IL-4, IL-10, VEGF, and platelet-activating factor (PAF)] was detected using the ELISA kits following the protocols.

### 2.10 RNA pull-down assay

DSCR9 was synthesized using GS ™ The T7 Biotin Labeled RNA Synthesis Kit (Guangzhou Geneseed Biotech Co., Ltd., Guangzhou, China) as per the manufacturer’s instructions. The interaction between DSCR9 and RPLP2 was detected using the PureBindingTM RNA Protein pull-down Kit (Geneseed) following the manufacturer’s instructions. The RNA binding protein complex was washed, eluted, and then subjected to silver staining analysis. Afterward, Western blot was used to verify DSCR9 and RPLP2, following the same steps as before.

### 2.11 5-Ethynyl-2′-deoxyuridine staining

The co-cultured RA-FLSs were seeded onto 96-well plates and incubated with EdU diluent. Next, the cells were fixed with 4% paraformaldehyde for 30 min and then washed with phosphate-buffered saline (PBS). Cell proliferation was detected using the EdU staining reagent (RiboBio, Guangdong, China). DAPI was employed for nuclear staining, and then cells were observed under a fluorescence microscope.

### 2.12 Preparation of XFC drug-containing serum

A total of 30 male Sprague-Dawley (SD) rats were randomly allocated into two groups: the normal serum group (n = 10) and the XFC drug-containing serum group (n = 20). According to previous studies (36), rats in the XFC drug-containing group received a gavage of XFC suspension (0.648g/100g/d) for 7 consecutive days, while rats in the normal serum group were orally administered physiological saline (2mL/100g/d) for 7 consecutive days. One hour after the final administration, rats were anesthetized with pentobarbital sodium (50mg/kg). Rat blood was collected from the abdominal aorta into coagulation tubes and then centrifuged (3000 rpm, 10 min) for serum isolation. The serum was then incubated and inactivated in a water bath (56°C, 30 min), filtered through a 0.22 μm membrane, and stored at -80°C for subsequent analysis. All animal procedures in this study were approved by the Animal Ethics Committee of Anhui University of Traditional Chinese Medicine (AHUCM rats 2023020).

### 2.13 Animals and experimental protocols

A total of 40 male SD rats (200 ± 20 g; Jiangsu Yuxi Oriental Breeding Co., Ltd) were cultured (18-22°C, 40-60% humidity). All rats were healthy and had free access to food and water. An AA model of rats was established as described in previous literature (38). Rats were randomly allocated into 4 groups (n = 10): normal (treated with daily oral gavage of 2mL/100g physiological saline), model (treated with daily oral gavage of 2mL/100g physiological saline), XFC (treated with 0.12g/100g XFC suspension), and TPL groups (treated with 0.1mg/100g TPL solution). Rats in each group were administrated by daily gavage at a dose of 2 mL/100g for 4 consecutive weeks (39). At the end of the experiment, rats were euthanized via intraperitoneal injection of pentobarbital sodium (60-100 mg/kg), and their blood was collected from the abdominal aorta for serum isolation.

Additionally, rat knee joints were excised and fixed in 4% paraformaldehyde for pathological assessment. After dehydration with gradient alcohol, clearance, and paraffin embedding, the samples were sectioned (4 μm). The sections then underwent hematoxylin and eosin (HE) staining to evaluate synovial hyperplasia, inflammatory cell infiltration, and pannus formation under light microscopy. A scoring scale ranging from 0 to 4 was adopted in this study, as reported in previous research (68). For ultrastructural analysis, synovial tissue from the rat knee joints was promptly dissected into 1 mm³ blocks, fixed, dehydrated, and prepared according to standard protocols. After ultra-thin sectioning and staining, the synovial cell ultrastructure was observed under a transmission electron microscope (TEM).

### 2.14 Statistical analysis

Statistical analysis and charting of experimental data were conducted using IBM SPSS Statistics 22 and GraphPad Prism 8.2 software. Quantitative data that conformed to a normal distribution were represented as mean ± standard deviation and analyzed using a *t*-test or one-way analysis of variance. Quantitative data that did not follow a normal distribution were represented as quartiles [M (Q25, Q75)] and analyzed using non-parametric tests. The Spearman correlation method was employed for correlation analysis. A *p*-value of < 0.05 indicated a statistically significant difference.

## 3 Results

### 3.1 XFC could reduce clinical inflammation and coagulation indicators, and improve SPP in RA patients

The changes in SPP and clinical laboratory indicators before and after XFC treatment were compared and analyzed. According to the results, compared with those before treatment, regarding the SPP indicators, SAS, SDS, and VAS were significantly decreased, and PF, RP, BP, GH, VT, SF, RE, and MH of RA patients were increased after treatment; clinical indicators (FBG, DD, ESR, and Hs-CRP) were remarkably decreased (**Table 2**). These results indicated that XFC can reduce clinical inflammation and coagulation indicators in RA patients, and improve SPP of RA patients.

**Table 2:** Changes in clinical inflammatory indicators, coagulation indicators, and SPP before and after treatment.

Indicator	Pre-treatment (n=151)	Post-treatment (n=151)	*p*-value
SAS	58 (53, 63)	45 (37, 51)	<0.001
SDS	63 (58, 68)	50 (45, 55)	<0.001
PF	15 (0, 30)	40 (25, 55)	<0.001
RP	0 (0, 0)	0 (0, 50)	<0.001
BP	31 (22, 41)	51 (41, 62)	<0.001
GH	25 (15, 33)	35 (25, 45)	<0.001
VT	35 (25, 45)	50 (40, 60)	<0.001
SF	38 (25, 50)	50 (38, 63)	<0.001
RE	0 (0, 33)	0 (0, 67)	<0.001
MH	40 (32, 48)	52 (46, 64)	<0.001
VAS	5.0 (5.0, 6.0)	2.0 (1.0, 3.0)	<0.001
PLT	240 (184, 302)	244 (186, 313)	0.586
FBG	4.05 (3.19, 5.06)	3.54 (3.09, 4.44)	0.007
DD	0.93 (0.47, 2.60)	0.54 (0.32, 1.83)	0.032
ESR	32 (15, 55)	16 (7, 37)	<0.001
Hs-CRP	13 (4, 45)	2 (1, 7)	<0.001
RF	117 (39, 232)	97 (34, 180)	0.245
CCP	105 (20, 332)	89 (19, 281)	0.679

### 3.2 XFC treatment was closely related to the improvement of clinical inflammatory indicators, coagulation indicators, and SPP in RA patients

**Figure 1:**
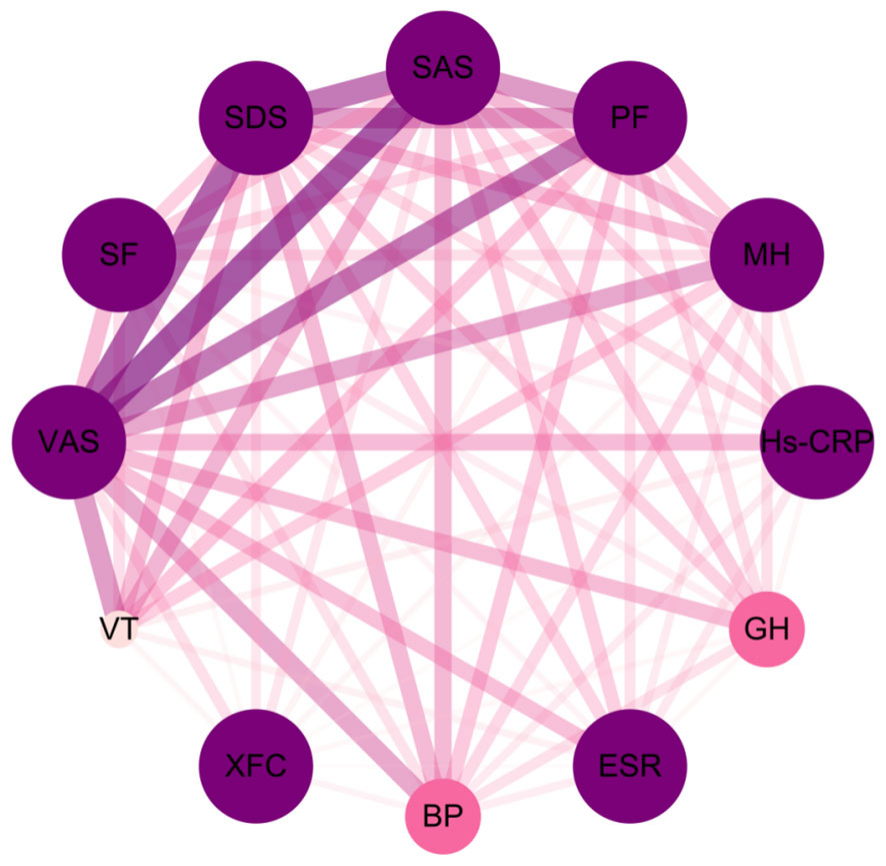
Complex network diagram of association rules analysis between XFC and clinical inflammation indicators, coagulation indicators, and SPP indicators in RA. The thickness of the connecting line and the depth of the color are positively correlated with the indicators.

Through association rule analysis of clinical data of RA patients, it was found that there is a strong correlation (lift>1) between the use of XFC and the reduction of VAS, SAS, SDS, Hs-CRP, and ESR, as well as the increase of PF, BP, MH, VT, GH, and SF (**Table 3**). The complex network diagram showed a strong correlation between XFC treatment and the improvement of inflammation indicators, coagulation indicators, and SPP (**Figure 1**). The above results further indicated that the use of XFC can reduce clinical inflammation and coagulation indicators in RA patients, and improve their SPP.

**Table 3:** Analysis of association rules between XFC and clinical inflammatory, coagulation, and SPP indicators in RA.

Consequent	Antecedent	Support (%)	Confidence (%)	Lift
VAS↓	XFC	46.32	98.01	1.021
SAS↓	XFC	46.32	92.72	1.099
SDS↓	XFC	46.32	92.05	1.132
PF↑	XFC	46.32	78.15	1.048
BP↑	XFC	46.32	77.48	1.226
MH↑	XFC	46.32	74.17	1.157
VT↑	XFC	46.32	74.17	1.125
GH↑	XFC	46.32	71.52	1.247
SF↑	XFC	46.32	68.87	1.207
Hs-CRP↓	XFC	46.32	65.56	1.125
ESR↓	XFC	46.32	62.25	1.068

### 3.3 PI3K/AKT was tightly implicated in XFC treatment of RA

**Figure 2:**
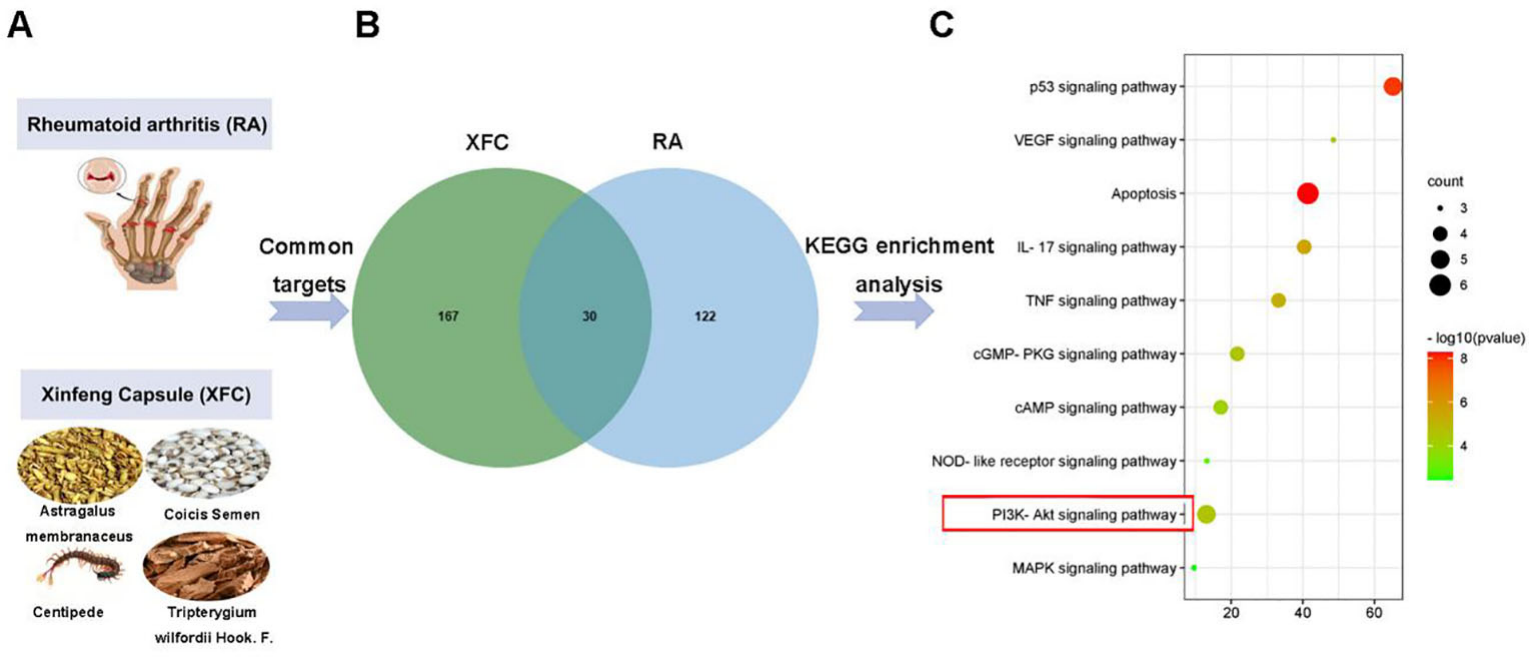
PI3K/AKT was tightly implicated in XFC treatment of RA. **(A)** The image of RA and the composition of XFC. **(B)** Related targets of XFC and RA. **(C)** Relevant pathways for XFC’s effects on RA.

XFC is composed of 4 TCM herbs (**Figure 2A**). Based on the pharmacokinetic characteristics of OB ≥ 30% and DL ≥ 0.18, 92 potential active ingredients and 197 targets were screened in the TCMSP database. Additionally, a total of 3577 RA-related targets were collected through the GeneCards, OMIM, DrugBank, and TTD databases. After removing the duplicate genes, 152 RA-related targets were finally obtained. Subsequently, Venn analysis was performed on 197 targets and 152 RA-related targets corresponding to XFC active ingredients using the RData software. As a result,30 overlapping genes were identified, which were potential targets for XFC treatment of RA (**Figure 2B**). KEGG pathway enrichment analysis was conducted,and the top 10 signaling pathways were selected for visual analysis based on the magnitude of the P-value. As revealed by the KEGG enrichment analysis results, PI3K/AKT was one of the key pathways for XFC treatment of RA (**Figure 2C**).

### 3.4 LncDSCR9 was correlated with clinical inflammatory coagulation indicators and SPP-related indicators

**Figure 3:**
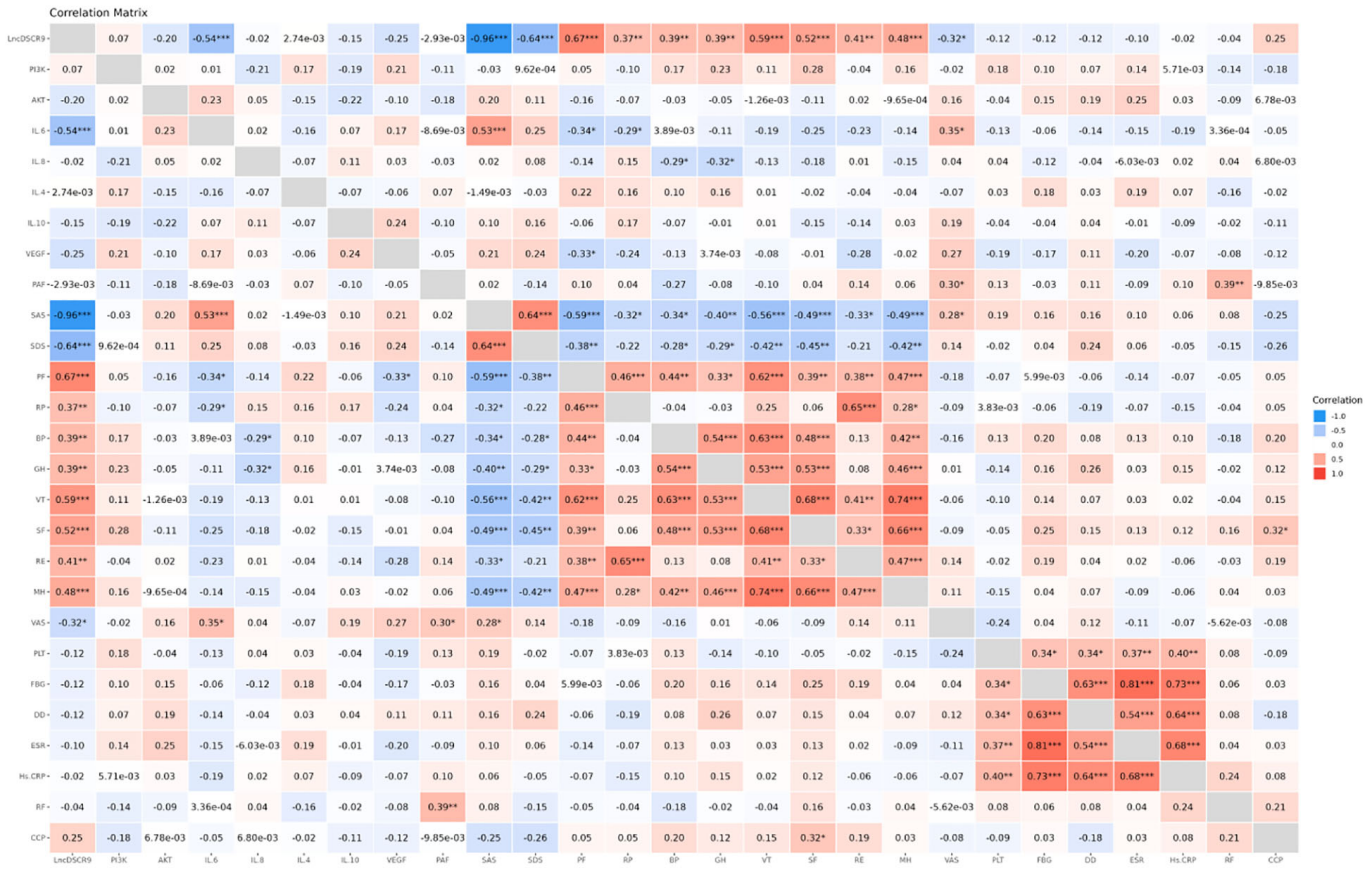
LncDSCR9 was correlated with clinical inflammatory coagulation indicators and SPP-related indicators. *p < 0.05, **p < 0.01, ***p < 0.001.

According to Spearman correlation analysis results, lncRNA DSCR9 was positively correlated with RP, BP, GH, MH, PF, SF, RE, and VT levels, and negatively correlated with SAS, SDS, and VAS levels. IL-6 was positively correlated with VAS and SAS levels, and negatively correlated with PF and RP levels. IL-8 was negatively correlated with BP and GH levels. PAF was positively correlated with RF and VAS levels. VEGF was negatively correlated with PF levels. Additionally, PLT was positively correlated with Hs-CRP, ESR, DD, and FBG levels. Similarly, FBG was positively correlated with Hs-CRP, ESR, and DD levels. DD was positively correlated with Hs-CRP and ESR levels (**Figure 3**; **Supplementary Tables S1**, **S2**). These results suggested that the inflammation in RA patients was closely related to hypercoagulability; lncRNA DSCR9, inflammation indicators, and hypercoagulability indicators were tightly related to SPP in RA patients.

### 3.5 XFC treatment affected the lncRNA DSCR9/PI3K/AKT axis in RA-PBMCs

**Figure 4:**
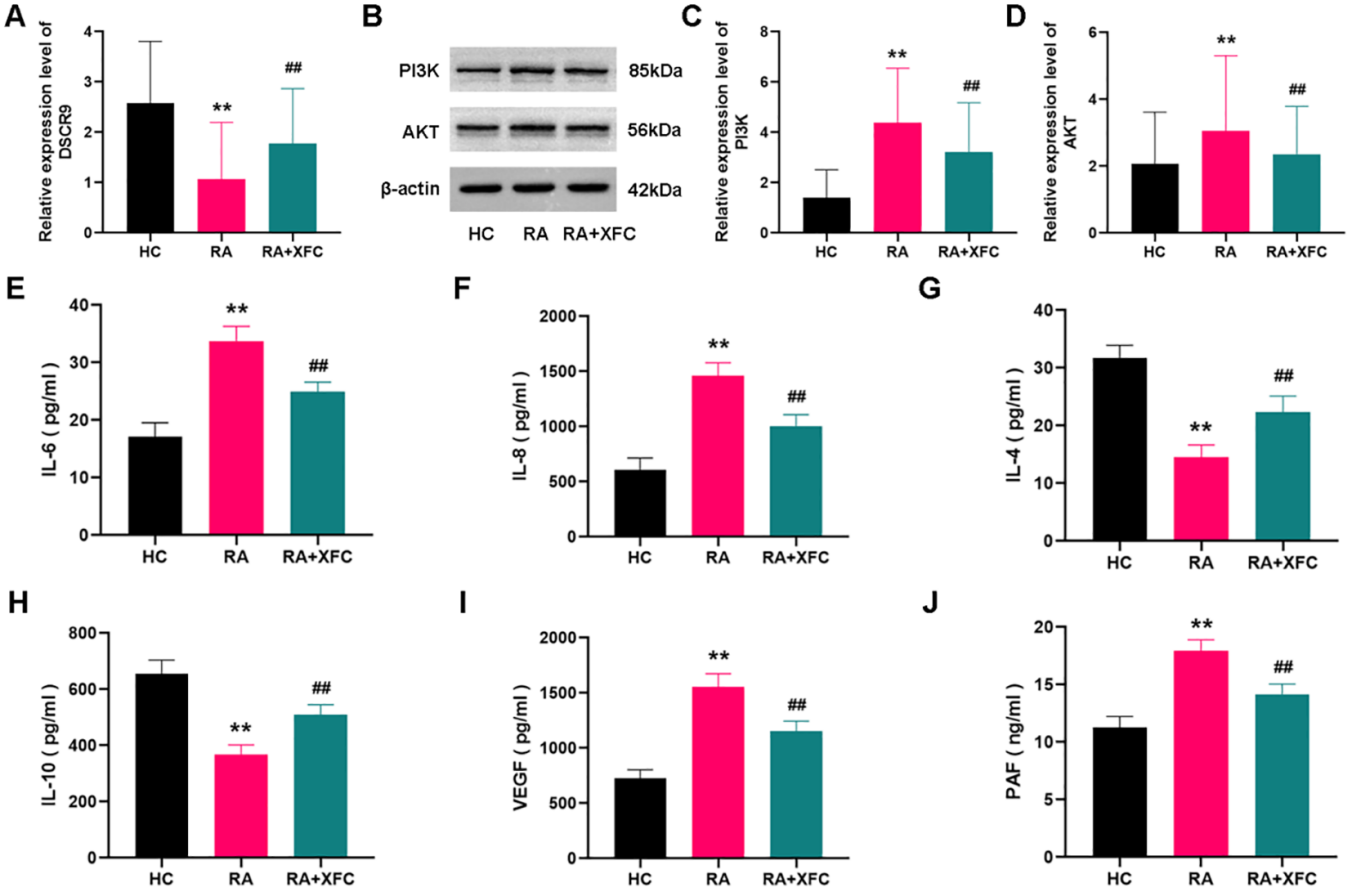
XFC treatment affected the expression of lncRNA DSCR9/PI3K/AKT and inflammatory/coagulation cytokines in RA-PBMCs. HC, healthy control group (control group); RA, rheumatoid arthritis group (before treatment); RA+XFC, XFC treatment group (after treatment). **(A)** Analysis of lncRNA DSCR9 expression through RT-qPCR. **(B–D)** Detection of PI3K and AKT protein levels through Western blot. **(E-J)** Evaluation of IL-6, IL-8, IL-4, IL-10, VEGF, and PAF levels through ELISA. **p < 0.01, compared to the HC group; ##*p* < 0.01, compared to the RA group.

Subsequently, the potential roles of lncRNA DSCR9/PI3K/AKT and inflammatory/coagulation cytokines in RA progression were investigated, A total of 30 healthy control (HC) subjects and 50 RA patients were enrolled, and no statistical difference between the two groups was found in terms of age and gender (*p* > 0.05, **Table 4**). Expressions of lncRNA DSCR9, PI3K, AKT, IL-6, IL-8, IL-4, IL-10, VEGF, and PAF were examined before and after XFC treatment in both groups. LncRNA DSCR9 expression was decreased, while PI3K and AKT expression levels were increased in PBMCs of RA patients. However, RT-qPCR detection results demonstrated that lncRNA DSCR9 showed an opposite expression trend in the XFC treatment group (**Figure 4A**). Western blot analysis also confirmed opposite results of PI3K and AKT levels after XFC treatment compared to those before treatment (**Figures 4B-D**). The levels of inflammation and coagulation factors were detected using ELISA. It was found that IL-6, IL-8, VEGF, and PAF levels were increased, while IL-4 and IL-10 levels were decreased in RA patients. On the contrary, XFC treatment could reverse the above results (**Figures 4E-J**).

**Table 4:** Basic information of healthy volunteers and RA patients.

Indicator	HC (n=30)	RA (n=50)	p-value
Gender (Male/Female)	4/26	3/47	0.416
Age (years)	58 ± 9	62 ± 11	0.093
Disease duration (years)	NA	10 (5, 20)	NA

NA, Not applicable.

Therefore, these results suggested that lncRNA DSCR9, PI3K, AKT, IL-6, IL-8, IL-4, IL-10, VEGF, and PAF were involved in RA progression. Among them, the expression of lncRNA DSCR9, IL-4, and IL-10 was decreased in RA-PBMCs and increased after XFC treatment. In contrast, the expression of PI3K, AKT, IL-6, IL-8, VEGF, and PAF was upregulated in RA-PBMCs, while effectively decreased after XFC treatment.

### 3.6 Overexpression of lncRNA DSCR9 can inhibit inflammation and hypercoagulability in RA

**Figure 5:**
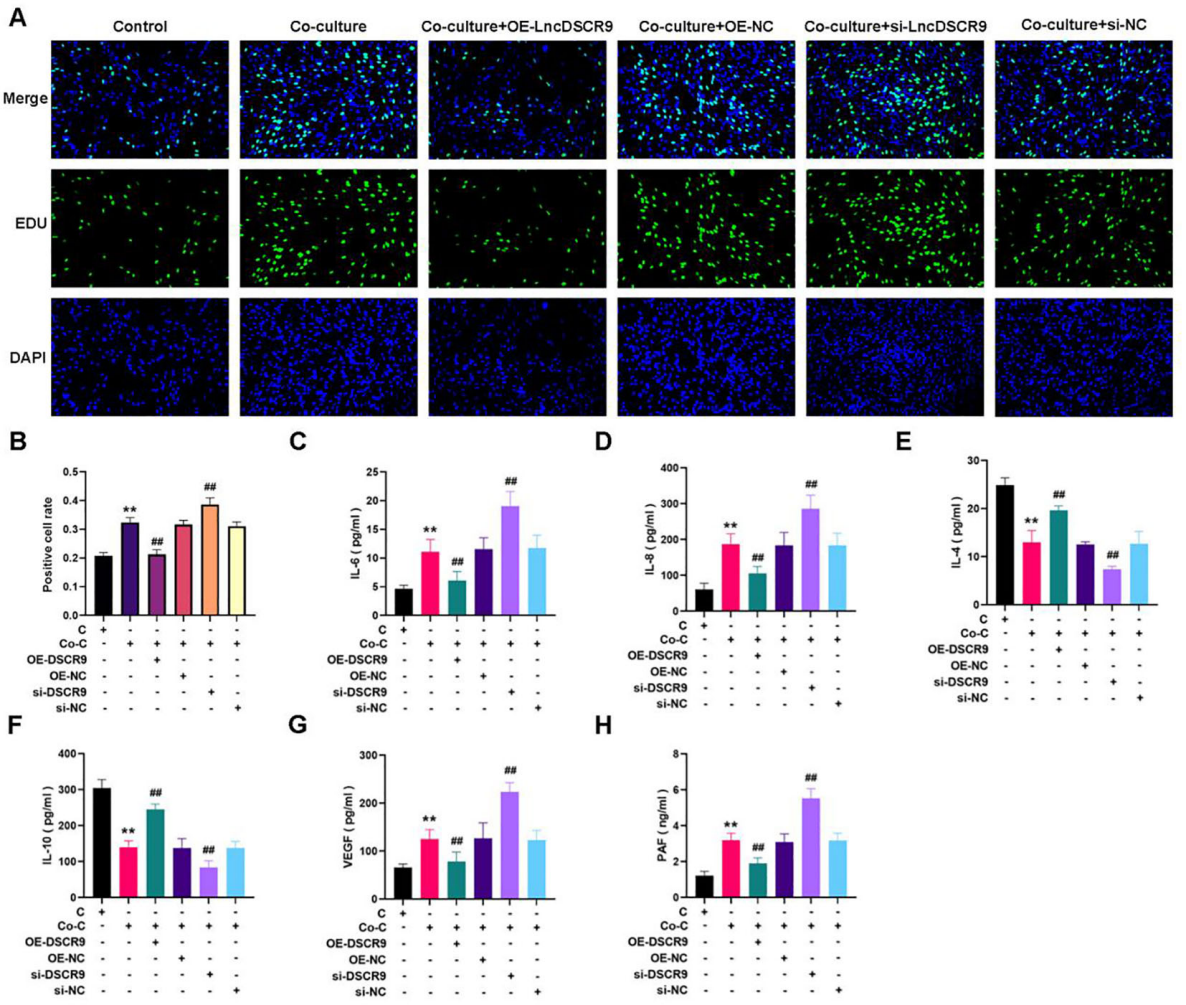
Overexpression of lncRNA DSCR9 weakened the inflammatory response and hypercoagulable state in RA. **(A)** Detection of cell proliferation using EdU staining. **(B)** Detection of positive cell rate using EdU staining. **(C-H)** Quantification of IL-6, IL-8, IL-4, IL-10, VEGF, and PAF using ELISA. *p < 0.05, **p < 0.01 relative to the control group. #*p* < 0.05, ##*p* < 0.01, relative to the co-culture group. C, Control group (RA-FLSs); Co-C, Co-culture group (co-culture of RA-PBMCs and RA-FLSs).

A co-culture model of RA PBMCs and RA-FLSs was established to demonstrate the effects of lncRNA DSCR9 on RA inflammation and hypercoagulability. The optimal cell ratio between RA-PBMCs and RA-FLSs was initially determined to be 2.5:1, and the optimal co-culture time was 48 h (40). According to EdU results, si-DSCR9 promoted the proliferation, while OE-DSCR9 inhibited the proliferation of co-cultured RA-FLSs (**Figures 5A, B**). ELISA results showed that IL-6, IL-8, VEGF, and PAF levels were increased, while IL-4 and IL-10 levels were decreased in the si-DSCR9 group. The OE-DSCR9 group showed opposite results, as manifested by the decreased levels of IL-6, IL-8, VEGF, and PAF (**Figures 5C, D, G, H**), and increased levels of IL-4 and IL-10 (**Figures 5E, F**). These findings indicated that knocking out DSCR9 could drive the inflammatory response and hypercoagulable state of RA, while overexpression of DSCR9 can weaken the inflammatory response and hypercoagulable state.

### 3.7 RPLP2 was the target of lncDSCR9

**Figure 6:**
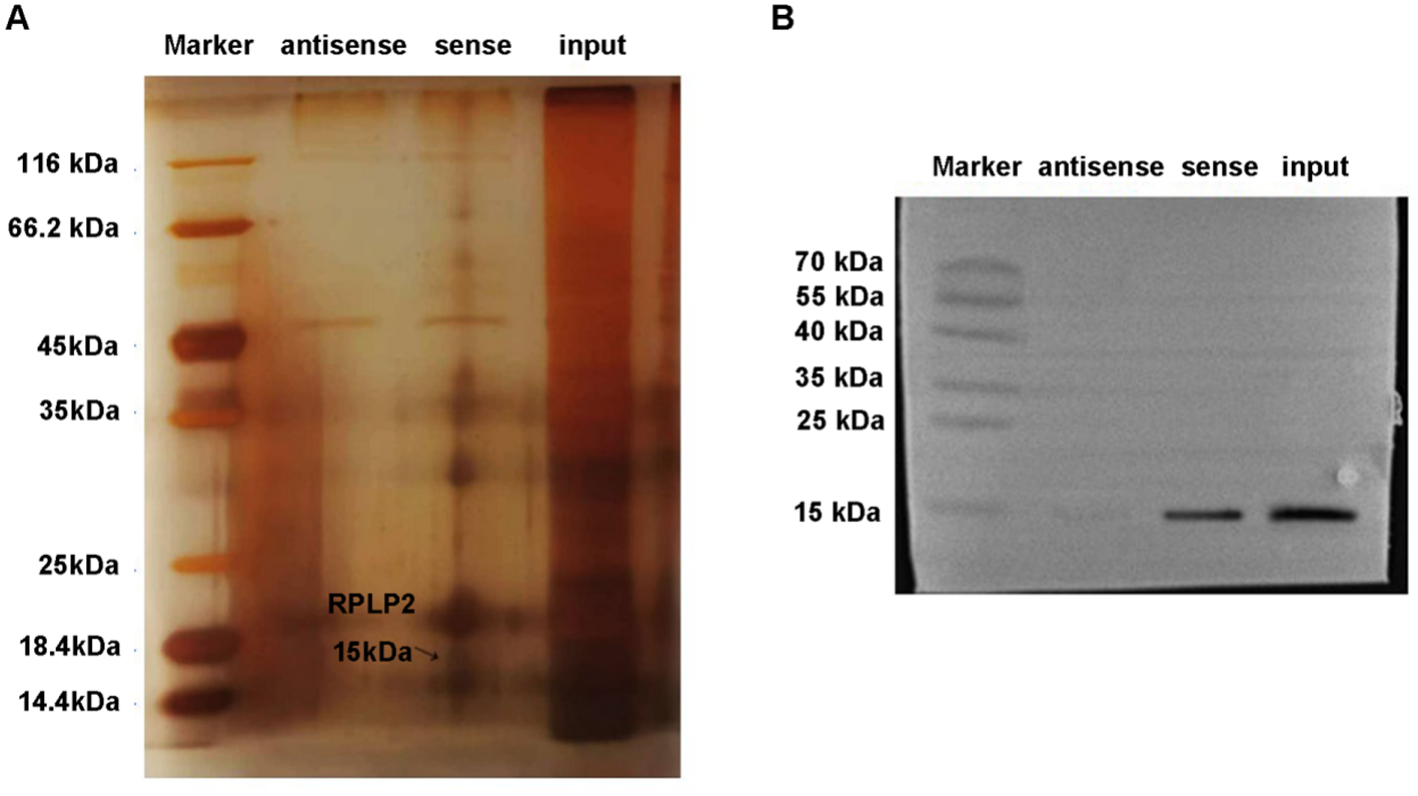
RPLP2 was the target of LncDSCR9. **(A)** Protein silver staining results. Identification of differentially expressed proteins pulled down by lncRNA DSCR9 using mass spectrometry, with RPLP2 indicated by arrows. **(B)** Western blot validation of the binding between DSCR9 and RPLP2.

Subsequently, the action mechanism of lncRNA DSCR9 was further explored and its target was clarified. LncRNA DSCR9 was converted to ENSG000000230366 through the Ensemble database (https://asia.ensembl.org/index.html). Then, the target gene of lncRNA DSCR9 was predicted to be RPLP2 through the Starbase gene library (https://starbase.sysu.edu.cn/index.php). Moreover, through the IntaRNA database (https://rna.informatik.uni-freiburg.de/IntaRNA/Input.jsp?JobID=5890431&reload=true), it’s determined that lncRNA DSCR9 and RPLP2 had binding sites, with a binding energy of -12.44 kcal/mol. Mass spectrometry analysis was further utilized to identify the differentially expressed proteins pulled down by lncRNA DSCR9, and the target RPLP2 then was revealed (**Figure 6A**). Additionally, the binding of lncRNA DSCR9 to RPLP2 was verified using Western blot analysis (**Figure 6B**). Therefore, lncRNA DSCR9 may act through RPLP2.

### 3.8 LncDSCR9 inhibited the PI3K/AKT pathway activation by downregulating RPLP2 to improve inflammation and hypercoagulability

**Figure 7:**
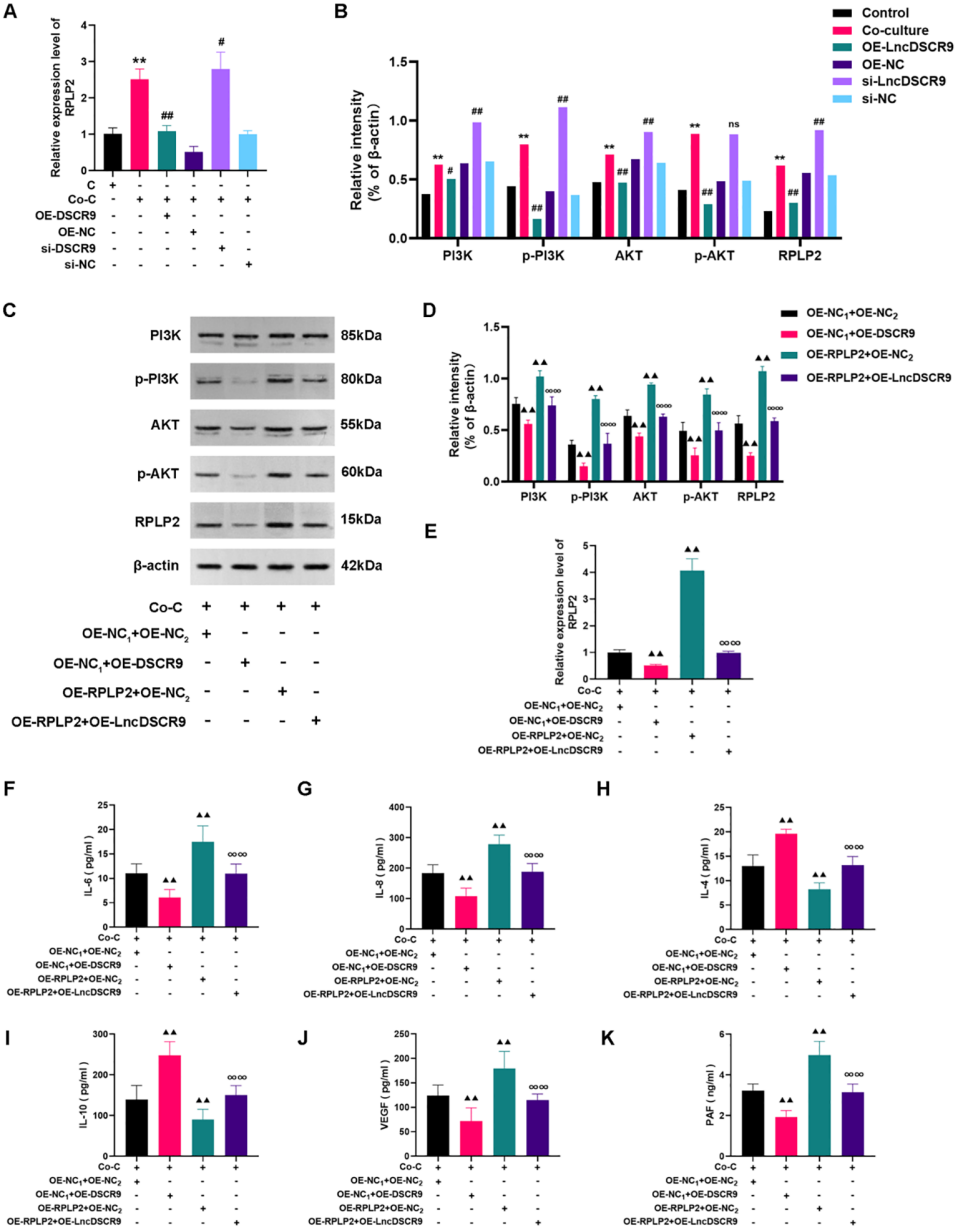
LncDSCR9 inhibited the PI3K/AKT pathway activation by downregulating RPLP2 to improve inflammation and hypercoagulability. **(A)** and **(B)** Effects of knockdown/overexpression of lncRNA DSCR9 on RPLP2, PI3K, AKT, p-PI3K, and p-AKT expressions. **(C–E)** Effects of transfection with OE-RPLP2 and OE-DSCR9 on PI3K, AKT, p-PI3K, p-AKT, and RPLP2 levels. **(F–K)** Effects of transfection with OE-RPLP2 and OE-DSCR9 on IL-6, IL-8, IL-4, IL-10, VEGF, and PAF levels. **p < 0.01, relative to the control group. #*p* < 0.05, ##*p* < 0.01, relative to the co-culture group. ▴▴*p*< 0.01, relative to the co-culture group. ∞∞*p* < 0.01, relative to the OE-RPLP2+OE-NC_2_ group. Cm Control group (RA-FLSs); Co-C, Co-culture group (co-culture of RA-PBMCs and RA-FLSs).

To elucidate the mechanism of lncRNA DSCR9 regulating RA inflammation and hypercoagulability, we then detected the expression of RPLP2/PI3K/AKT in a co-culture model of RA-PBMCs and RA-FLSs. Additionally, the levels of inflammation and coagulation indicators were also evaluated after OE-RPLP2 transfection. The RT-qPCR results revealed that RPLP2 expression was increased in the si-DSCR9 group, while decreased in the OE-DSCR9 group (**Figure 7A**). As indicated by Western blot results, RPLP2, PI3K, AKT, p-PI3K, and p-AKT protein levels were increased in the si-DSCR9 group, while decreased in the OE-DSCR9 group (**Figure 7B**). After OE-DSCR9 treatment, RPLP2 expression and PI3K, AKT, p-PI3K, and p-AKT levels in the OE-RPLP2 group were decreased (**Figure 7C-E**). Similarly, OE-DSCR9 treatmentcould decrease IL-6, IL-8, IL-4, IL-10, VEGF, and PAF levels, while increase IL-4 and IL-10 levels in the OE-RPLP2 group (**Figures 7F-K**). These results indicated that lncRNA DSCR9 inhibited the PI3K/AKT pathway activation by downregulating RPLP2, thereby improving inflammation and hypercoagulability.

### 3.9 The PI3K/AKT pathway activation promoted inflammation and hypercoagulability in co-cultured RA-FLSs

**Figure 8:**
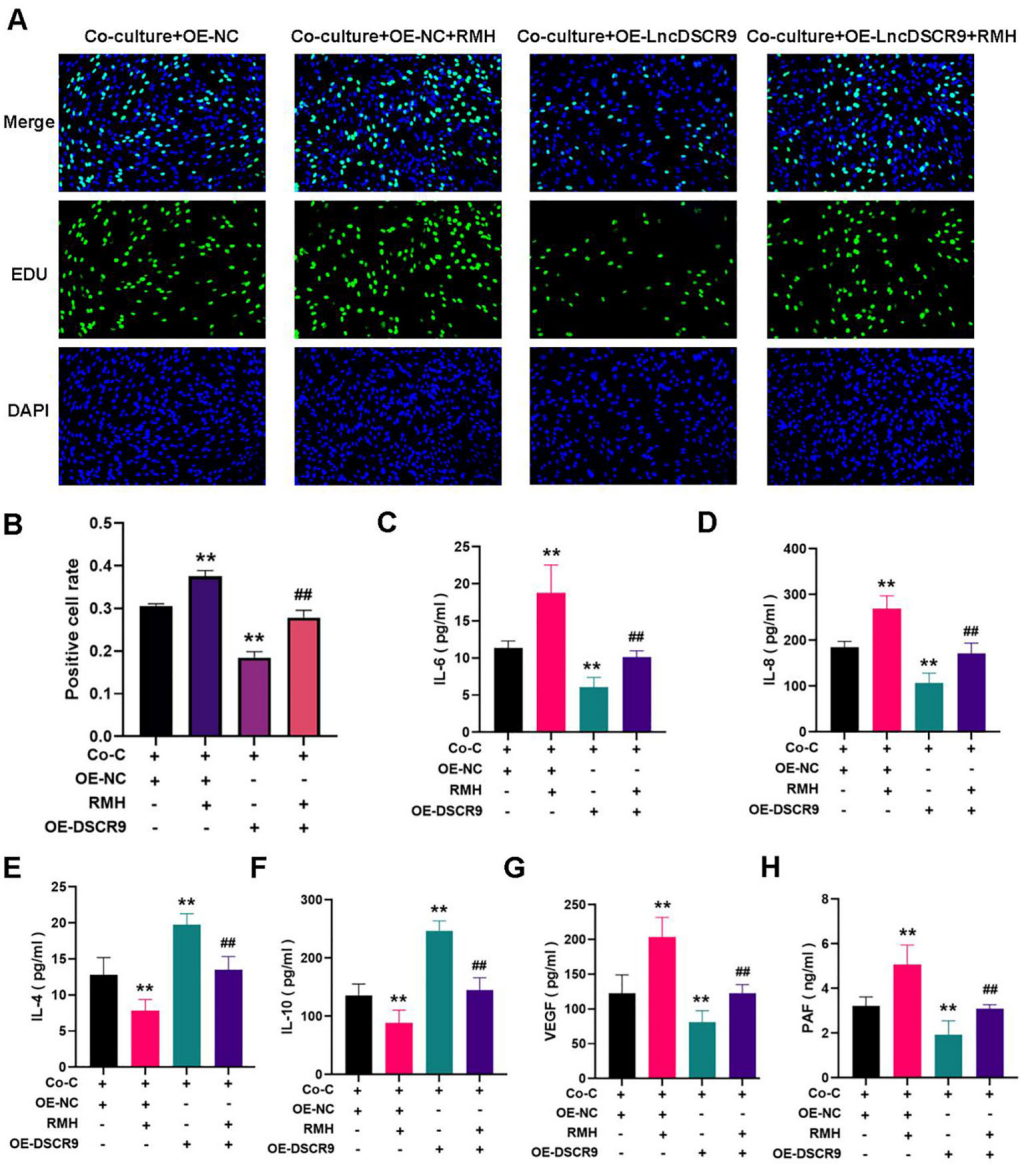
Activation of the PI3K/AKT pathway promoted inflammation and hypercoagulability in co-cultured RA-FLSs. **(A)** Detection of cell proliferation using EdU staining. **(B)** Detection of positive cell rate using EdU staining. **(C-H)** Effects of transfection with DSCR9 and the addition of RMH on IL-6, IL-8, IL-4, IL-10, VEGF, and PAF levels. **p < 0.01, relative to co-culture + OE-NC group. ##*p* < 0.01, relative to the co-culture + OE-lncDSCR9 group. Co-C, Co-culture group (co-culture of RA-PBMCs and RA-FLSs).

Whether the PI3K/AKT pathway was involved in the regulation of lncRNA DSCR9 in RA-FLSs was further explored.As PI3K is located upstream of AKT, and activating PI3K can also activate AKT, PI3K activator (RMH) was added in this study. The EdU staining results revealed that RMH can promote the proliferation of co-cultured RA-FLSs and reverse the inhibitory effect of OE-LncDSCR9 on co-cultured RA-FLS proliferation (**Figures 8A, B**). Additionally, the ELISA results showed that RMH can increase IL-6, IL-8, VEGF, and PAF levels, reduce IL-4 and IL-10 levels, and reverse the effect of OE-LncDSCR9 on these cytokines (**Figures 8C-H**). These above results indicated that the PI3K/AKT pathway activation promoted the inflammatory response and hypercoagulable state of co-cultured RA-FLSs.

### 3.10 XFC promoted lncRNA DSCR9 expression, inhibited RPLP2 and PI3K/AKT expressions, and suppressed the production of pro-inflammatory/pro-coagulant cytokines

The effects of different concentrations (5%, 10%, 20%, and 50%) of XFC drug-containing serum on the viability of co-cultured RA-FLS viability were detected using the CCK-8 assay at 24 h, 48 h, and 72 h, respectively. It was found that at 48 h, XFC drug-containing serum at 10% concentration had an inhibition rate of reaching 50% on co-cultured RA-FLSs. Therefore, 10% XFC-containing serum was selected as the optimal concentration, and 48 h was chosen as the optimal treatment time for the subsequent experiments (40).

**Figure 9:**
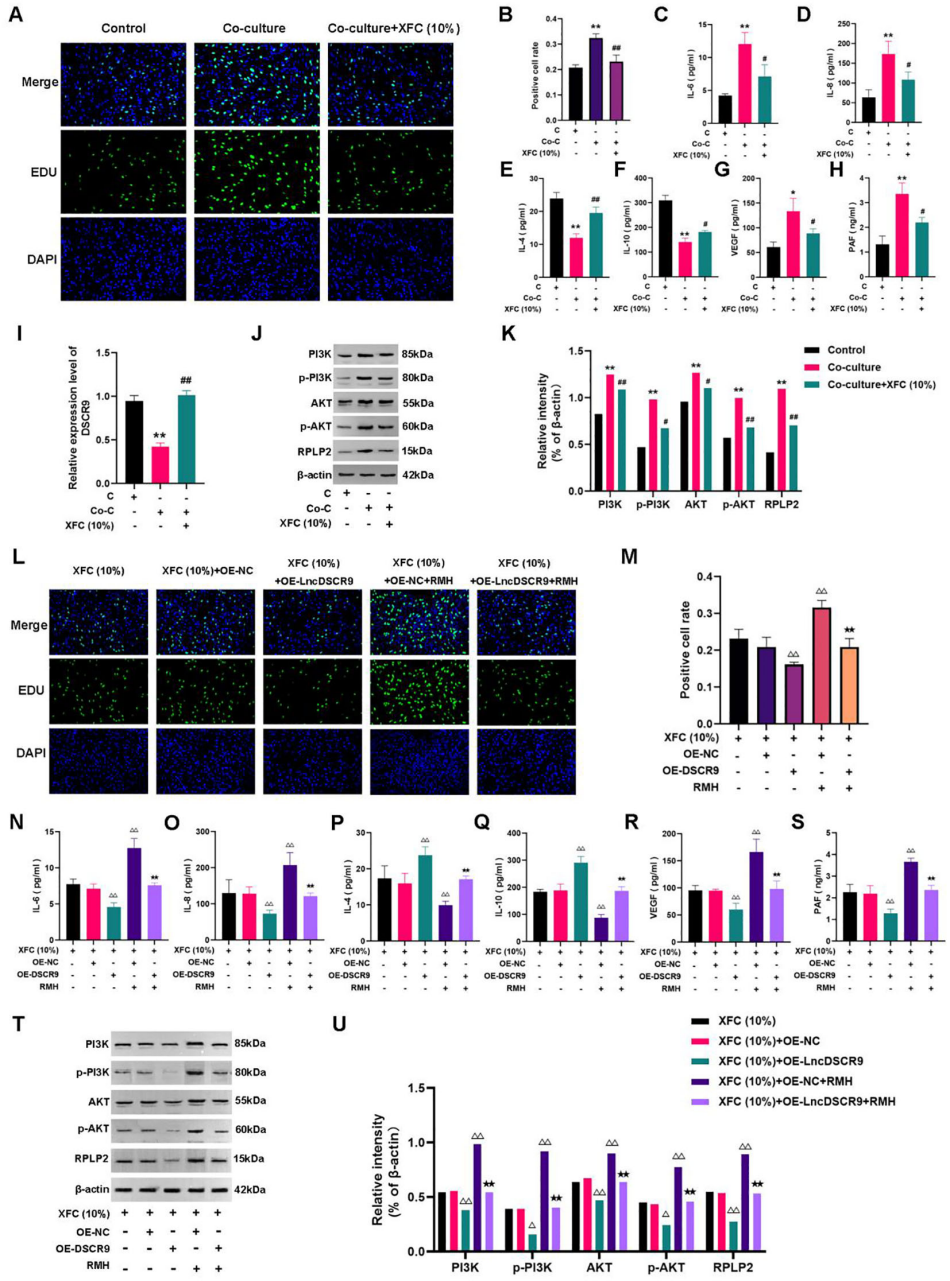
XFC inhibited inflammation and hypercoagulability of RA-FLSs through the LncDSCR9/RPLP2/PI3K/AKT axis. **(A)** Detection of cell proliferation using EdU staining. **(B)** Detection of positive cell rate using EdU staining. **(C-H)** Detection of the effects of XFC (10%) on IL-6, IL-8, IL-4, IL-10, VEGF, and PAF expression levels. **(I-K)** Detection of the effects of XFC (10%) on DSCR9, RPLP2, PI3K, AKT, p-PI3K, and p-AKT levels. **(L)** Detection of cell proliferation using EdU staining. **(M)** Detection of positive cell rate using EdU staining. **(N-S)** Detection of the effects of XFC (10%) on IL-6, IL-8, IL-4, IL-10, VEGF, and PAF levels with the addition of OE-DSCR9 and RMH. **(T)** and **(U)** Detection of the effects of XFC (10%) on PI3K, AKT, p-PI3K, p-AKT, and RPLP2 levels with the addition of OE-DSCR9 and RMH. *p < 0.05, **p < 0.01, relative to the control group. #*p* < 0.05, ##*p* < 0.01, relative to the co-culture group. △*p* < 0.05, △△*p*< 0.01, relative to the XFC (10%) + OE-NC group. ★*p* < 0.05, ★★*p* < 0.01, relative to the XFC (10%) + OE-NC + RMH group. C, Control group (RA-FLSs); Co-C, Co-culture group (co-culture of RA-PBMCs and RA-FLSs); XFC (10%), XFC-containing serum at 10% concentration.

XFC (10%) can inhibit the proliferation of co-cultured cells (**Figures 9A, B**), and suppress IL-6, IL-8, VEGF, and PAF levels while increasing IL-4 and IL-10 levels (**Figures 9C-H**). Additionally, XFC treatment increased lncRNA DSCR9 expression and decreased the expression levels of PI3K, AKT, p-PI3K, p-AKT, and RPLP2 (**Figures 9I-K**). Taken together, XFC (10%) exhibited similar therapeutic effects to OE-DSCR9 in inhibiting PI3K/AKT-induced inflammation and hypercoagulability.

### 3.11 RMH can antagonize the effect of XFC on the co-cultured RA-FLSs

Functional response experiments were further conducted to investigate whether XFC improved inflammation and hypercoagulability through the PI3K/AKT pathway. The EdU results showed that RMH antagonized the inhibitory effect of XFC on the proliferation of co-cultured RA-FLSs (**Figures 9L, M**). Similar results were also observed in ELISA and Western blot analysis results. In RMH-treated co-cultured RA-FLSs, IL-6, IL-8, VEGF, PAF, PI3K, AKT, p-PI3K, p-AKT, and RPLP2 expression levels were upregulated, while IL-4 and IL-10 levels were downregulated (**Figures 9N-U**). These results indicated that RMH can antagonize the therapeutic effect of XFC on the co-cultured RA-FLSs.

### 3.12 OE-DSCR9 enhanced the effect of XFC on the co-cultured RA-FLSs

Finally, the rescue experiments were conducted by incorporating OE-DSCR9. The EdU results indicated that compared with the XFC (10%) + OE-NC group,the XFC (10%) + OE-DSCR9 group showed inhibited cell proliferation, and OE-DSCR9 reversed the promoting effect of RMH on cell proliferation (**Figures 9L, M**). ELISA results showed that IL-6, IL-8, VEGF, and PAF levels were remarkably reduced, while IL-4 and IL-10 levels were increased in the XFC (10%) + OE-DSCR9 group compared with those in the XFC (10%) + OE-NC group (**Figures 9N-S**). The cytokine expression trend in the XFC (10%) + RMH + OE-DSCR9 group was the same as that in the XFC (10%) + RMH group. According to Western blot analysis results, compared with those in the XFC (10%) + OE-NC group, PI3K, p-PI3K, AKT, p-AKT, and RPLP2 levels in the XFC (10%) + OE-DSCR9 group were remarkably reduced; the protein level trend in the XFC (10%) + RMH + OE-DSCR9 was the same as that in the XFC (10%) + RMH group (**Figures 9T, U**). Taken together, OE-DSCR9 can synergize with XFC to inhibit the inflammation and hypercoagulability of co-cultured RA-FLSs induced by the PI3K/AKT pathway activation. In summary, XFC can inhibit the inflammatory response and hypercoagulable state of co-cultured RA-FLSs through the LncDSCR9/RPLP2/PI3K/AKT axis.

### 3.13 XFC alleviated inflammation and hypercoagulability in AA rats

**Figure 10:**
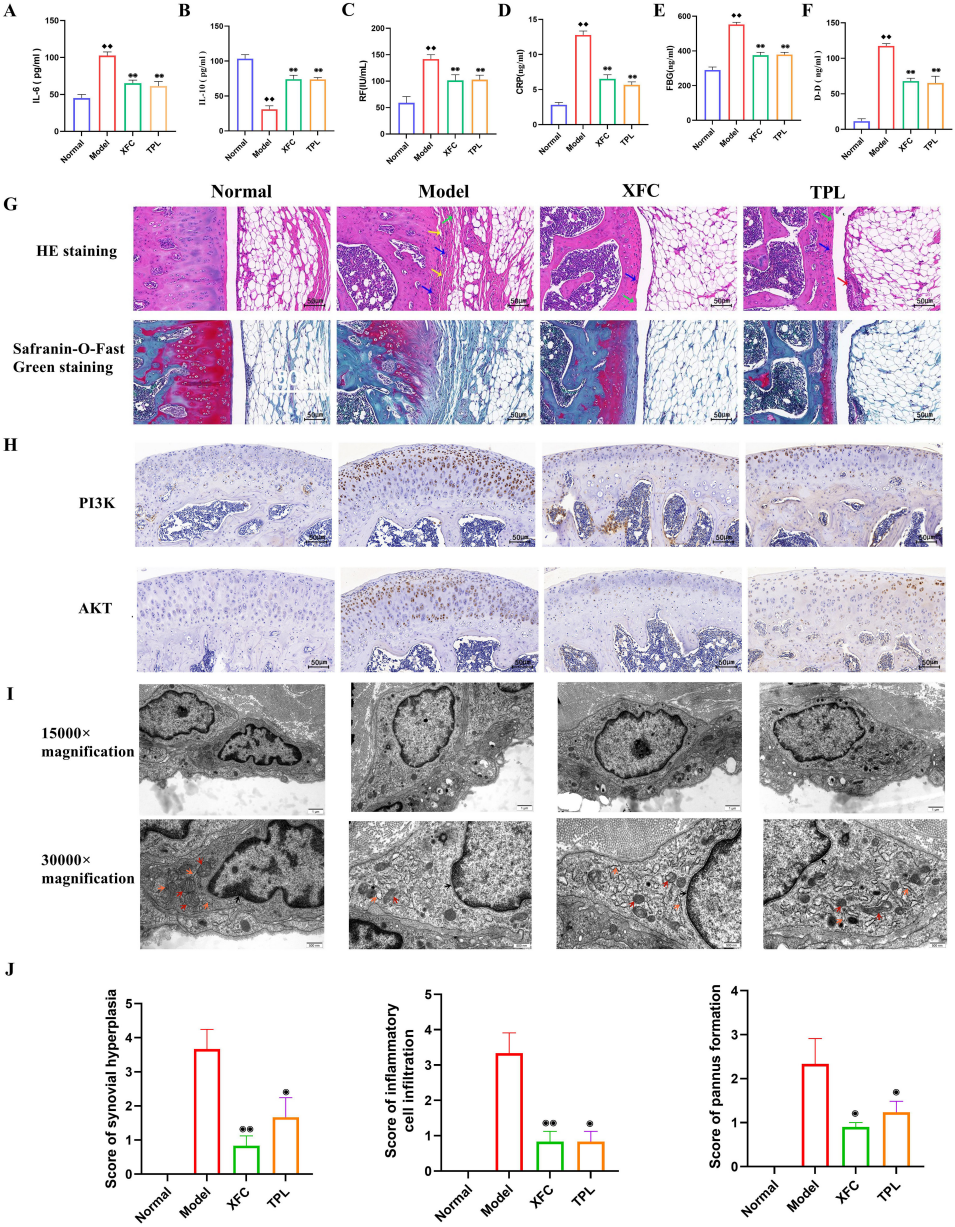
XFC alleviated the inflammatory response and hypercoagulable state in AA rats. **(A-F)** Detection of RF, CRP, IL-6, IL-10, FBG, and DD levels by ELISA. **(G)** Detection of the effect of XFC on the pathological changes of knee joint tissue in AA rats using HE staining and Safranin-O-Fast Green staining (red arrows indicated synovial epithelial hyperplasia, green arrows indicated cartilage surface fibrosis, blue arrows indicated inflammatory cell infiltration, and yellow arrows indicated articular cartilage destruction and bone erosion). **(H)** Detection of the expression of PI3K and AKT proteins in the synovium of AA rats using immunohistochemical staining. **(I)** Observation of the effect of XFC on the ultrastructure of synovial cells in AA rats under TEM (arrow pointing to changes in organelles and mitochondria). **(J)** Assessment of pathological scores for synovial hyperplasia, inflammatory cell infiltration, and pannus formation using HE staining. ♦♦*p* < 0.01, relative to the normal group. ⦿*p* < 0.05, ⦿⦿*p* < 0.01, relative to the model group. Model group: AA rats.

Previous studies have confirmed that XFC can effectively improve tissue swelling and arthritis index in AA rats (41). For further evaluating whether XFC can alleviate inflammation and hypercoagulability in AA rats, RA diagnostic indicators (RF and CRP), inflammatory factors (IL-6and IL-10), and coagulation indicators (FBG and DD) were detected. The pathological and ultrastructural changes in AA rats were also observed. The results showed that compared with those in the normal group, the serum levels of RF, CRP, IL-6, FBG, and DD in AA rats were notably increased, while IL-10 level was remarkably reduced (**Figures 10A-F**). This indicated that the inflammation and hypercoagulability were promoted and anti-inflammatory ability was inhibited in AA rats. After XFC and TPL treatment, the above indicators were effectively improved (**Figures 10A-F**).

According to HE staining results, AA rats showed synovial tissue proliferation, accompanied by extensive infiltration of inflammatory cells, narrow joint space, uneven joint surface, and pannus formation. All these were consistent with the basic pathological characteristics of human RA (**Figure 10G**). Safranin-O-Fast Green staining results revealed disrupted knee joint structure, damaged bone integrity, synovial tissue-invaded cartilage, degraded and damaged red cartilage matrix, and rough surface of the joint cartilage in AA rats (**Figure 10G**). After XFC and TPL treatment, the surface of the knee joint in rats was relatively intact, and the degradation of the cartilage matrix was reduced (*p* < 0.05, **Figure 10J**). This indicated that XFC efficiently inhibited synovial hyperplasia, inflammatory cell infiltration, and cartilage destruction in AA rats.

As indicated by immunohistochemical staining results, there were brownish-yellow granular substances (PI3K and AKT) in the cells (**Figure 10H**). The treatment with XFC and TPL reduced the expression of PI3K and AKT, suggesting that XFC may exert anti-inflammatory and anticoagulant effects through the PI3K/AKT pathway. TEM observed that the number of organelles in synovial cells of the model group rats was significantly reduced, accompanied by mitochondrial vacuolar degeneration, and mitochondrial cristae rupture or even disappearance. There was also significant expansion and damage to the rough endoplasmic reticulum in the model rats. After XFC and TPL treatments, the nuclear membrane boundaries of synovial cells were clear, with mild mitochondrial swelling, clear mitochondrial cristae, and normal morphology of the rough endoplasmic reticulum (**Figure 10I**). The above results indicated that XFC can improve the inflammatory response and hypercoagulable state of AA rats.

## 4 Discussion

RA is a chronic, progressive, and invasive autoimmune disorder characterized by synovitis and extra-articular manifestations (42), which has posed a significant threat to patients’ quality of life. If left untreated, RA can result in disability, early retirement, and even premature mortality (43). RA’s complications not only involve joint deterioration and malformation, but a broad spectrum of organs, including the cardiovascular system, lungs, eyes, kidneys, and skeletal system, leading to severe socio-economic burdens and high mortality rates (44). In addition to the intricate immune-inflammatory cascade that characterizes RA’s onset, the role of hypercoagulability in RA progression has also garnered increasing attention (45). XFC, a proprietary hospital preparation from Anhui Provincial Hospital of Traditional Chinese Medicine, has been utilized for RA management for a long time (46). Research has demonstrated that XFCcan mitigate RA-related inflammation, improve hypercoagulability, and alleviate joint pain (16). However, the underlying mechanisms remain largely unexplored. This study investigated the mechanisms underlying the therapeutic effects of XFC on inflammation and hypercoagulability in RA, aiming to provide evidence for elucidating the pathogenesis of RA and the potential targeted drug therapies, with a focus on clinical translation and application.

Studies have highlighted that RA patients often experience varying degrees of emotional and psychological fluctuations, manifesting as both psychological and physiological impairments, which significantly affect their daily life quality (8, 9). In response to these findings, this study conducted a comprehensive SPP assessment through retrospective data mining, encompassing 8 core domains of SF-36 (GH, PF, RE, MH, RP, BP, SF, and VT), alongside VAS, SAS, and SDS indicators. Our findings underscored that the SPP status was generally poor in RA patients, accompanied by increased clinical inflammatory and coagulation indicators. Intriguingly, XFC administration in RA patients can notably reduce these inflammatory and coagulation indicators, thereby significantly improving their SPP. The in-depth examination of RA clinical samples revealed a close correlation between inflammatory and coagulation biomarkers, as well as a significant link between lncRNA DSCR9 expression, inflammatory and coagulation biomarkers, and SPP changes in RA patients. These results implied that SPP fluctuations in RA patients maybe partially attributed to the inflammatory response and hypercoagulable state in the body. The subsequent clinical trials involving XFC intervention further validated the therapeutic potential of XFC in mitigating inflammation and hypercoagulability in RA patients, reinforcing its role as a promising treatment option for RA.

The inflammatory responses and a hypercoagulable state are intricately implicated in RA pathogenesis. FLSs are the main constituents of synovial cells, and can secret large amounts of inflammatory cytokines during the active phase of RA, exacerbating disease progression (47). Additionally, stimulation of the inflammatory cascade can trigger abnormal proliferation of RA-FLSs, resulting in the production of various inflammatory cytokines, which mutually promote each other and form a vicious circle (48). Within this cytokine milieu, IL-6 and IL-8 are multifunctional cytokines secreted by diverse cells, which can serve as pivotal inflammatory regulators with pro-inflammatory properties (49). Conversely, IL-4 is sourced from CD8+ T cells, and IL-10 is produced by monocytes, macrophages, B cells, and Th2 cells; both exhibit anti-inflammatory effects and can suppress the synthesis and activity of pro-inflammatory mediators and collaborate synergistically with other anti-inflammatory agents (50, 51). This study also uncovered an imbalance in the inflammatory cytokine network, showing elevated IL-6 and IL-8 levels and reduced IL-4 and IL-10 levels in RA patients, which was found to be intimately related to increasing disease activity indicators and decreasing SPP. Therefore, the unbalanced inflammatory cytokine network may play a crucial role in RA pathogenesis, and regulating this balance is of great value in treating RA. Furthermore, the pathological mechanisms of RA include intra-articular angiogenesis and pannus formation, which are also critical processes in the erosion of articular cartilage and bone. It has been evidenced that the imbalance of inflammatory factors (such as IL-10 and IL-6) is implicated in aberrant NF-κB factors, leading to a hypercoagulable state in RA (17). Additionally, PAF is a potent phospholipid neurotransmitter that can promote platelet aggregation, increase blood viscosity, and trigger thrombosis, further complicating the disease landscape (52). VEGF is a pivotal upstream signaling molecule and an effective modulator of angiogenesis, playing a crucial role in vascular-related bone formation and repair processes (53). Our clinical observations revealed that RA patients exhibited imbalances in both inflammatory and coagulation cytokines, with a notable association between inflammatory cytokines and SPP indicators. Furthermore, a significant correlation between clinical inflammatory indicators and hypercoagulability indicators was identified, underscoring the intricate interplay between the inflammatory and hypercoagulable states in RA patients. These findings collectively highlighted the interconnected nature of these two pathological conditions in the context of RA.

The co-cultivation of RA-PBMCs and RA-FLSs can reflect the pathological and physiological characteristics of RA. PBMCs, comprising lymphocytes and monocytes, collectively form a vital pillar of the immune system. RA is an autoimmune disorder, with its mechanism involving intricate humoral and cellular immunity. Research has illuminated that non-integrated laminin receptors on PBMCs can diminish lipopolysaccharide levels and stimulate TNF-α production (54). Comparative studies between RA patients and healthy individuals have revealed that the Ras-specific guanosine nucleotide release factor in T and B lymphocytes of RA patients is hindered, which fosters CD4+T lymphocyte proliferation and subsequently enhances B lymphocyte activity (55). Regulatory T lymphocytes have the main functions of inhibiting excessive immune activation and preserving immune homeostasis, and they may undergo quantitative, functional, and activational alterations in RA, contributing to immune dysregulation, inflammation, and joint deterioration (56). In summary, PBMCs play a multifaceted role in RA immunopathogenesis, and their modulation of inflammation and autoimmune responses may have significant implications for disease progression and treatment. Therefore, studying the function of PBMCs in RA is of great significance for a deeper understanding of disease mechanisms and the development of novel therapeutic avenues. Moreover, the pathological basis of RA lies in synovitis and pannus development. RA-FLSs are key effector cells in RA pathogenesis, predominantly located within the synovial intima and playing a significant role in disease progression (57). It has been shown that aberrant RA-FLS proliferation triggers inflammation and exacerbates synovial damage. These cells secrete a large amount of cytokines, chemokines, and matrix metalloproteinases, promoting inflammation and joint destruction (58). Additionally, RA-FLSs exhibit invasive properties, which can invade and damage joint cartilage and bone tissue, ultimately resulting in joint deterioration and deformity (59). Since the 1980s, researchers have gradually emphasized the significance of multifactorial and multicellular interactions in disease occurrence and development. Therefore, relying solely on a single cell line for disease studies is inadequate. Consequently, techniques for cell co-culture have emerged as a solution. TCM holds the holistic perspective that the human body is a whole, underscoring the interconnectedness and mutual influence among bodily components. Aligning with this view, cell co-culture simulates *in vivo* cellular coordination and interplay. This approach facilitates a deeper understanding of disease mechanisms, contributing to the study of intercellular signaling, mutual influence, and cellular function regulation (60). In this study, RA-PBMCs (representative of the systemic immune response) withwere co-cultured with RA-FLSs, (indicative of local pathological alterations) using the Transwell system. This approach mirrored the pathological and physiological features of RA while embodying the holistic view of TCM. Our findings revealed that stimulation of RA-PBMCs enhanced RA-FLS proliferation, increased the release of pro-inflammatory and pro-coagulant cytokines, and reduced the secretion of anti-inflammatory cytokines. This phenomenon could be attributed to the inflammatory/hypercoagulable microenvironment shaped by PBMCs secreting various messenger RNAs and cytokines.

Several investigations have highlighted the potential of lncDSCR9 as a biomarker for various cancers, including breast cancer, renal cell carcinoma, and pancreatic cancer (61–63). The current study observed that lncDSCR9 expression was reduced in RA patients, negatively associated with clinical inflammatory and coagulation markers but positively correlated with SF-36 scores. Notably, after XFC treatment, the expression of lncDSCR9 and proinflammatory/procoagulant cytokines was decreased, accompanied by an increase in anti-inflammatory cytokines. These findings indicated the therapeutic potential of lncDSCR9 in RA. For elucidating lncDSCR9’s mechanism in modulating inflammation and hypercoagulability in RA, a pull-down assay coupled with mass spectrometry was employed, and RPLP2 was identified as the target of lncDSCR9. Our findings suggested that lncDSCR9 downregulated RPLP2 expressionto inhibit the PI3K/AKT pathway, thereby improving inflammatory and hypercoagulable states. A previous study has pointed out that lncDSCR9 is implicated in PI3K/AKT signaling, platelet aggregation, and blood coagulation (21). The PI3K/AKT pathway is a pivotal regulator of RA-FLSs proliferation, apoptosis, and immune-inflammatory responses (64). Moreover, the PI3K/AKT pathway is intricately linked to microvascular injury and angiogenesis, with its overactivation promoting neovascularization and vessel expansion, a hallmark of RA-associated hypercoagulability (27). These findings further reinforced that RA-FLSs displayed excessive activation of the PI3K/AKT signaling pathway, contributing to inflammation and abnormal regulation of hypercoagulable cytokines. Nevertheless, XFC treatment effectively upregulated lncDSCR9 expression and downregulated RPLP2 expression, thus suppressing the PI3K/AKT pathway. XFC treatment also modulated cytokine profiles, as manifested by the suppressed IL-6, IL-8, VEGF, and PAF levels while enhanced IL-10 and IL-4 levels. Subsequently, the role of the lncDSCR9/RPLP2/PI3K/AKT axis in mediating XFC’s anti-inflammatory and anti-hypercoagulant effects was further explored through response and rescue experiments. In short, DSCR9 was overexpressed and the PI3K/AKT agonist RMH was employed, and the results confirmed that RMH countered XFC’s inhibitory effects on the co-cultured RA-FLSs. Conversely, OE-DSCR9 collaborated with XFC to synergistically inhibit the inflammation and hypercoagulability triggered by the PI3K/AKT pathway activation. These discoveries underscored the multi-faceted inhibitory action of XFC on inflammation and hypercoagulability, which is consistent with previous research endeavors. This study highlighted the potential of targeting this intricate signaling network for therapeutic intervention in RA.

The AA rat, as a classic animal model in the field of immune-inflammatory research, has been widely adopted for the screening of RA drugs and the in-depth investigation of their underlying mechanisms (65). Given this, an AA rat model was established using the induction method using Complete Freund’s Adjuvant (FCA). Further observations revealed that rats in the AA model group exhibited significant pathological features such as abnormal synovial hyperplasia, extensive infiltration of inflammatory cells, cartilage tissue damage, and angiogenesis, which are consistent with previous relevant studies (66). Notably, the serum levels of RF, CRP, IL-6, FBG, and DD were elevated, while the anti-inflammatory factor IL-10 was reduced in AA rats. These alterations were consistent with the inflammatory responses and coagulation status observed in RA patients. Subsequently, after XFC treatment, these biochemical indicators were markedly improved, suggesting a substantial therapeutic potential of XFC in the AA rat model. Synovial hyperplasia and inflammatory cell infiltration are the pathological basis of RA (57, 67). In this study, histopathological assessment visually demonstrated that XFC treatment effectively mitigated synovial hyperplasia, reduced inflammatory cell infiltration, and protected articular cartilage from further damage in AA rats. Additionally, TEM observation further unveiled the positive restorative effects of XFC on the ultrastructure of synovial cells, specifically manifested by significantly alleviated mitochondrial and rough endoplasmic reticulum damage. Furthermore, XFC reduced PI3K and AKT protein levels in the synovium of AA rats, indicating that XFC may inhibit the PI3K/AKT signaling pathway activation, thereby blocking the pathological cascade leading to synovial hyperplasia, inflammation exacerbation, and cartilage damage. Taken together, the therapeutic efficacy of XFC in the AA rat model may be intimately related to its regulation of the PI3K/AKT pathway.

## 5 Conclusion

In summary, our research findings confirm that the therapeutic effect of XFC on RA is mediated by the lncDSCR9/RPLP2/PI3K/AKT axis, which reduces inflammatory response and improves hypercoagulable state. Therefore, the present study provides evidence that XFC intervention is a new strategy for clinical treatment of RA.

## Data Availability

The original contributions presented in the study are included in the article/**Supplementary Material**. Further inquiries can be directed to the corresponding author.

## References

[1] Sparks JA. Rheumatoid arthritis. Ann Intern Med. (2019) 170:Itc1–itc16. doi: 10.7326/AITC201901010 30596879

[2] Smolen JS Aletaha D Barton A Burmester GR Emery P Firestein GS. Rheumatoid arthritis. Nat Rev Dis Primers. (2018) 4:18001. doi: 10.1038/nrdp.2018.1 29417936

[3] De Stefano L D'Onofrio B Manzo A Montecucco C Bugatti S. The genetic, environmental, and immunopathological complexity of autoantibody-negative rheumatoid arthritis. Int J Mol Sci. (2021) 22:12386. doi: 10.3390/ijms222212386 34830268 PMC8618508

[4] Payet M Dargai F Gasque P Guillot X. Epigenetic regulation (Including micro-RNAs, DNA methylation and histone modifications) of rheumatoid arthritis: A systematic review. Int J Mol Sci. (2021) 22:12170. doi: 10.3390/ijms222212170 34830057 PMC8625518

[5] Smolen JS Landewé RBM Bijlsma JWJ Burmester GR Dougados M Kerschbaumer A. et al: EULAR recommendations for the management of rheumatoid arthritis with synthetic and biological disease-modifying antirheumatic drugs: 2019 update. Ann rheum Dis. (2020) 79:685–99. doi: 10.1136/annrheumdis-2019-216655 31969328

[6] Rutherford AI Patarata E Subesinghe S Hyrich KL Galloway JB. Opportunistic infections in rheumatoid arthritis patients exposed to biologic therapy: results from the British Society for Rheumatology Biologics Register for Rheumatoid Arthritis. Rheumatology. (2018) 57:997–1001. doi: 10.1093/rheumatology/key023 29529307

[7] Mueller AL Payandeh Z Mohammadkhani N Mubarak SMH Zakeri A Alagheband Bahrami A. Recent advances in understanding the pathogenesis of rheumatoid arthritis: new treatment strategies. Cells. (2021) 10:3017. doi: 10.3390/cells10113017 34831240 PMC8616543

[8] Houge IS Hoff M Videm V. The association between rheumatoid arthritis and reduced estimated cardiorespiratory fitness is mediated by physical symptoms and negative emotions: a cross-sectional study. Clin Rheumatol. (2023) 42:1801–10. doi: 10.1007/s10067-023-06584-x PMC10038374 36964449

[9] Yıldırım Keskin A Şentürk S Kimyon G. Eating attitude in patients with rheumatoid arthritis: The relationship between pain, body mass index, disease activity, functional status, depression, anxiety and quality of life. Arch psychiat nurs. (2023) 44:52–8. doi: 10.1016/j.apnu.2023.04.001 37197863

[10] Croia C Bursi R Sutera D Petrelli F Alunno A Puxeddu I. One year in review 2019: pathogenesis of rheumatoid arthritis. Clin Exp Rheumatol. (2019) 37:347–57. 31111823

[11] Kubota A Suguro T Nakajima A Sonobe M Tsuchiya K. Effect of biological agents on synovial tissues from patients with rheumatoid arthritis. Mod Rheumatol. (2020) 30:282–6. doi: 10.1080/14397595.2019.1583783 30786801

[12] Nygaard G Firestein GS. Restoring synovial homeostasis in rheumatoid arthritis by targeting fibroblast-like synoviocytes. Nat Rev Rheumatol. (2020) 16:316–33. doi: 10.1038/s41584-020-0413-5 PMC7987137 32393826

[13] Korb-Pap A Bertrand J Sherwood J Pap T. Stable activation of fibroblasts in rheumatic arthritis-causes and consequences. Rheumatology. (2016) 55:ii64–7. doi: 10.1093/rheumatology/kew347 27856663

[14] Petrasca A Phelan JJ Ansboro S Veale DJ Fearon U Fletcher JM. Targeting bioenergetics prevents CD4 T cell-mediated activation of synovial fibroblasts in rheumatoid arthritis. Rheumatology. (2020) 59:2816–28. doi: 10.1093/rheumatology/kez682 32047926

[15] Sun H Zhang W Yang N Xue Y Wang T Wang H. Activation of cannabinoid receptor 2 alleviates glucocorticoid-induced osteonecrosis of femoral head with osteogenesis and maintenance of blood supply. Cell Death Dis. (2021) 12:1035. doi: 10.1038/s41419-021-04313-3 34718335 PMC8556843

[16] Wang FF Liu J Fang YY Wen JT He MY Han Q. Exploring the mechanism of action of xinfeng capsule in treating hypercoagulable state of rheumatoid arthritis based on data mining and network pharmacology. Natural Product Commun. (2022) 17:1934578X221119918. doi: 10.1177/1934578X221119918

[17] Zhang P Liu J Tan B Zhu F Fang L. Hypercoagulable state is associated with NF-kappa B activation and increased inflammatory factors in patients with rheumatoid arthritis. Xi Bao Yu Fen Zi Mian Yi Xue Za Zhi. (2016) 32:364–8. 26927557

[18] Di Gesualdo F Capaccioli S Lulli M. A pathophysiological view of the long non-coding RNA world. Oncotarget. (2014) 5:10976–96. doi: 10.18632/oncotarget.2770 PMC4294373 25428918

[19] Liu W Sheng L Nie L Wen X Mo X. Functional interaction between long non-coding RNA and microRNA in rheumatoid arthritis. J Clin Lab Anal. (2020) 34:e23489. doi: 10.1002/jcla.23489 33319382 PMC7755821

[20] Lao MX Xu HS. Involvement of long non-coding RNAs in the pathogenesis of rheumatoid arthritis. Chin Med j-peking. (2020) 133:941–50. doi: 10.1097/CM9.0000000000000755 PMC7176443 32187055

[21] Wen J Liu J Jiang H Wan L Xin L Sun Y. lncRNA expression profiles related to apoptosis and autophagy in peripheral blood mononuclear cells of patients with rheumatoid arthritis. FEBS Open Bio. (2020) 10:1642–54. doi: 10.1002/2211-5463.12913 PMC7396444 32569434

[22] Liu Z Mi M Li X Zheng X Wu G Zhang L. lncRNA OSTN-AS1 may represent a novel immune-related prognostic marker for triple-negative breast cancer based on integrated analysis of a ceRNA network. Front Genet. (2019) 10:850. doi: 10.3389/fgene.2019.00850 31572452 PMC6753250

[23] Chen M Wang J Luo Y Huang K Shi X Liu Y. Identify Down syndrome transcriptome associations using integrative analysis of microarray database and correlation-interaction network. Hum Genomics. (2018) 12:2. doi: 10.1186/s40246-018-0133-y 29351810 PMC5775600

[24] He J Lin X Wang X Lin T Lyu S Gao X. Arecoline hydrobromide suppresses PI3K/AKT pathway in rheumatoid arthritis synovial fibroblasts and relieves collagen-induced arthritis in mice. Int immunopharmacol. (2023) 124:110925. doi: 10.1016/j.intimp.2023.110925 37742366

[25] Li N Li X Deng L Yang H Gong Z Wang Q. 6-Shogaol inhibits the proliferation, apoptosis, and migration of rheumatoid arthritis fibroblast-like synoviocytes via the PI3K/AKT/NF-κB pathway. Phytomedicine. (2023) 109:154562. doi: 10.1016/j.phymed.2022.154562 36610124

[26] Jiang RH Xu JJ Zhu DC Li JF Zhang CX Lin N. Glycyrrhizin inhibits osteoarthritis development through suppressing the PI3K/AKT/NF-κB signaling pathway. Vivo vitro Food Funct. (2020) 11:2126–36. doi: 10.1039/c9fo02241d 32073014

[27] Domigan CK Ziyad S Iruela-Arispe ML. Canonical and noncanonical vascular endothelial growth factor pathways: new developments in biology and signal transduction. Arterioscl throm vas. (2015) 35:30–9. doi: 10.1161/ATVBAHA.114.303215 PMC4270848 25278287

[28] Roubenoff R Freeman LM Smith DE Abad LW Dinarello CA Kehayias JJ. Adjuvant arthritis as a model of inflammatory cachexia. Arthritis rheum-us. (1997) 40:534–9. doi: 10.1002/art.1780400320 9082942

[29] Song CY Xu YG Lu YQ. Use of Tripterygium wilfordii Hook F for immune-mediated inflammatory diseases: progress and future prospects. J zhejiang univ-sc B. (2020) 21:280–90. doi: 10.1631/jzus.B1900607 PMC7183448 32253838

[30] Fan D Guo Q Shen J Zheng K Lu C Zhang G. The effect of triptolide in rheumatoid arthritis: from basic research towards clinical translation. Int J Mol Sci. (2018) 19:376. doi: 10.3390/ijms19020376 29373547 PMC5855598

[31] Wen J Liu J Wang X Wang J. Triptolide promotes the apoptosis and attenuates the inflammation of fibroblast-like synoviocytes in rheumatoid arthritis by down-regulating lncRNA ENST00000619282. Phytother Res. (2021) 35:4334–46. doi: 10.1002/ptr.7129 34161642

[32] Gao LL Wang F Meng M. Chromatographic fingerprinting and quantitative analysis for the quality evaluation of Xinfeng capsule. Acta Chromatogr. (2021) 33:37–43. doi: 10.1556/1326.2020.00743

[33] Wang Y Liu J Wang Y Sun Y. Effect of Xinfeng capsule in the treatment of active rheumatoid arthritis: a randomized controlled trial. J tradit Chin Med. (2015) 35:626–31. doi: 10.1016/s0254-6272(15)30150-3 26742305

[34] Sun Y Liu J Xin L Wen J Zhou Q Chen X. Xinfeng capsule inhibits inflammation and oxidative stress in rheumatoid arthritis by up-regulating LINC00638 and activating Nrf2/HO-1 pathway. J Ethnopharmacol. (2023) 301:115839. doi: 10.1016/j.jep.2022.115839 36272490

[35] Wan L Liu J Huang CB Zhao L Chen X Fan HX. Mechanism of Xinfeng Capsules improving rheumatoid arthritis based on CD19~+B cells regulating FAK/CAPN/PI3K pathway. Zhongguo Zhong Yao Za Zhi. (2021) 46:3705–11. doi: 10.19540/j.cnki.cjcmm.20201120.501 34402295

[36] Fang Y Liu J Xin L Jiang H Wen J Li X. Xinfeng capsule inhibits lncRNA NONHSAT227927.1/TRAF2 to alleviate NF-κB-p65-induced immuno-inflammation in ankylosing spondylitis. J Ethnopharmacol. (2024) 323:117677. doi: 10.1016/j.jep.2023.117677 38160870

[37] Aletaha D Neogi T Silman AJ Funovits J Felson DT Bingham CO. 2010 rheumatoid arthritis classification criteria: an American College of Rheumatology/European League Against Rheumatism collaborative initiative. Ann rheum Dis. (2010) 69:1580–8. doi: 10.1136/ard.2010.138461 20699241

[38] Wan L Liu J Huang C Zhang W Qi Y Zhang X. Xinfeng Capsule improves lung function by regulating Notch/Jagged-HES axis of type II alveolar epithelial cells in adjuvant arthritis rats. Xi Bao Yu Fen Zi Mian Yi Xue Za Zhi. (2017) 33:942–6. 28712402

[39] Wan L Liu J Huang CB Chen X Wang Y Zhang WD. Effect of triptolide on expressions of Notch receptors and ligands in rats with adjuvant- induced arthritis and reduced pulmonary function. Nan Fang Yi Ke Da Xue Xue Bao. (2015) 35:1390–4. 26547329

[40] Wen JT. The Mechanism of LncRNA MAPKAPK5-AS1/miR-146a-3p/SIRT1 Combination Mediating Immunoinflammation and Apoptosis Escape in Rheumatoid Arthritis and Intervention of Xinfeng Capsule. Anhui, Anhui Province, China: Anhui University of Chinese Medicine (2022).

[41] Jiang H Liu J Gao J Meng M Qin X Wang T. Up-regulations of Bax and caspase-3 and down-regulation of Bcl-2 after Xinfeng capsule treatment in adjuvant-induced arthritis rats. Xi Bao Yu Fen Zi Mian Yi Xue Za Zhi. (2016) 32:457–61. 27053609

[42] Hao F Tao L Liu J Ma Y Zhang J Wang W. Cynanchum komarovii extract for the treatment of rheumatoid arthritis by acting on synovial cells. Vitro vivo J Ethnopharmacol. (2023) 317:116825. doi: 10.1016/j.jep.2023.116825 37348792

[43] Smolen JS Aletaha D Koeller M Weisman MH Emery P. New therapies for treatment of rheumatoid arthritis. Lancet. (2007) 370:1861–74. doi: 10.1016/S0140-6736(07)60784-3 17570481

[44] Alam J Jantan I Bukhari SNA. Rheumatoid arthritis: Recent advances on its etiology, role of cytokines and pharmacotherapy. BioMed pharmacother. (2017) 92:615–33. doi: 10.1016/j.biopha.2017.05.055 28582758

[45] Wang F Liu J Fang Y Wen J He M Han Q. Hypercoagulability in rheumatoid arthritis: A bibliometric analysis and retrospective data mining study. ACS Omega. (2023) 8:48522–34. doi: 10.1021/acsomega.3c08460 PMC10734003 38144152

[46] Zhang PH Liu J Tan B Wan L. Xinfeng capsule improved blood stasis state of rheumatoid arthritis patients based on actl/NF-KB signaling pathway: mechanism and effects. Zhongguo Zhong Xi Yi Jie He Za Zhi. (2016) 36:922–8. 30640985

[47] Umekita K Miyauchi S Nomura H Umeki K Okayama A. Neutrophil-derived lactoferrin induces the inflammatory responses of rheumatoid arthritis synovial fibroblasts via Toll-like receptor 4. Clin Exp Rheumatol. (2019) 37:834–41. 30767875

[48] Zhao Y Chen Y Wang J Zhu X Wang K Li Y. Ginkgolide J protects human synovial cells SW982 via suppression of p38−dependent production of pro−inflammatory mediators. Mol Med Rep. (2021) 24:555. doi: 10.3892/mmr.2021.12194 34080024 PMC8188640

[49] McInnes IB Schett G. Cytokines in the pathogenesis of rheumatoid arthritis. Nat Rev Immunol. (2007) 7:429–42. doi: 10.1038/nri2094 17525752

[50] Iwaszko M Biały S Bogunia-Kubik K. Significance of interleukin (IL)-4 and IL-13 in inflammatory arthritis. Cells. (2021) 10:3000. doi: 10.3390/cells10113000 34831223 PMC8616130

[51] Teng MW Darcy PK Smyth MJ. Stable IL-10: a new therapeutic that promotes tumor immunity. Cancer Cell. (2011) 20:691–3. doi: 10.1016/j.ccr.2011.11.020 22172716

[52] Mazereeuw G Herrmann N Xu H Blanchard AP Figeys D Oh PI. Platelet activating factors are associated with depressive symptoms in coronary artery disease patients: a hypothesis-generating study. Neuropsychiatr Dis Treat. (2015) 11:2309–14. doi: 10.2147/NDT.S87111 PMC4567245 26379437

[53] Yang C Wang J Chen L Xu T Ming R Hu Z. Tongluo Shenggu capsule promotes angiogenesis to ameliorate glucocorticoid-induced femoral head necrosis via upregulating VEGF signaling pathway. Phytomedicine. (2023) 110:154629. doi: 10.1016/j.phymed.2022.154629 36608500

[54] Kane BA An H Rajasekariah P McNeil HP Bryant K Tedla N. Differential expression and regulation of the non-integrin 37/67-kDa laminin receptor on peripheral blood leukocytes of healthy individuals and patients with rheumatoid arthritis. Sci Rep. (2019) 9:1149. doi: 10.1038/s41598-018-37907-7 30718719 PMC6362087

[55] Golinski ML Vandhuick T Derambure C Fréret M Lecuyer M Guillou C. Dysregulation of RasGRP1 in rheumatoid arthritis and modulation of RasGRP3 as a biomarker of TNFα inhibitors. Arthritis Res Ther. (2015) 17:382. doi: 10.1186/s13075-015-0894-9 26714738 PMC4718016

[56] Nakachi S Sumitomo S Tsuchida Y Tsuchiya H Kono M Kato R. Interleukin-10-producing LAG3+ regulatory T cells are associated with disease activity and abatacept treatment in rheumatoid arthritis. Arthritis Res Ther. (2017) 19:97. doi: 10.1186/s13075-017-1309-x 28511719 PMC5434528

[57] Bartok B Firestein GS. Fibroblast-like synoviocytes: key effector cells in rheumatoid arthritis. Immunol Rev. (2010) 233:233–55. doi: 10.1111/j.0105-2896.2009.00859.x PMC2913689 20193003

[58] Bottini N Firestein GS. Duality of fibroblast-like synoviocytes in RA: passive responders and imprinted aggressors. Nat Rev Rheumatol. (2013) 9:24–33. doi: 10.1038/nrrheum.2012.190 23147896 PMC3970924

[59] Xue M McKelvey K Shen K Minhas N March L Park SY. Endogenous MMP-9 and not MMP-2 promotes rheumatoid synovial fibroblast survival, inflammation and cartilage degradation. Rheumatology. (2014) 53:2270–9. doi: 10.1093/rheumatology/keu254 24982240

[60] Tang Y Wang B Sun X Li H Ouyang X Wei J. Rheumatoid arthritis fibroblast-like synoviocytes co-cultured with PBMC increased peripheral CD4+ CXCR5+ ICOS+ T cell numbers. Clin Exp Immunol. (2017) 190:384–93. doi: 10.1111/cei.13025 PMC5680054 28833034

[61] Liu Y Luo J Zeng J Liu W Fu B Xiong J. Competing endogenous RNA analysis identified lncRNA DSCR9 as a novel prognostic biomarker associated with metastasis and tumor microenvironment in renal cell carcinoma. Oncol Lett. (2023) 26:290. doi: 10.3892/ol.2023.13876 37274469 PMC10236252

[62] Huang H Li X Zhang X Li Z Han D Gao W. DSCR9/miR-21-5p axis inhibits pancreatic cancer proliferation and resistance to gemcitabine via BTG2 signaling. Acta bioch bioph Sin. (2022) 54:1775–88. doi: 10.3724/abbs.2022194 PMC10157615 36789695

[63] Li M Lin C Cai Z. Downregulation of the long noncoding RNA DSCR9 (Down syndrome critical region 9) delays breast cancer progression by modulating microRNA-504-5p-dependent G protein-coupled receptor 65. Hum Cell. (2023) 36:1516–34. doi: 10.1007/s13577-023-00916-4 37248366

[64] Yan X Liu Y Kong X Ji J Zhu H Zhang Z. MicroRNA-21-5p are involved in apoptosis and invasion of fibroblast-like synoviocytes through PTEN/PI3K/AKT signal. Cytotechnology. (2019) 71:317–28. doi: 10.1007/s10616-018-0288-3 PMC6368525 30599075

[65] Anchi P Swamy V Godugu C. Nimbolide exerts protective effects in complete Freund's adjuvant induced inflammatory arthritis via abrogation of STAT-3/NF-κB/Notch-1 signaling. Life Sci. (2021) 266:118911. doi: 10.1016/j.lfs.2020.118911 33333049

[66] Wang G Xie X Yuan L Qiu J Duan W Xu B. Resveratrol ameliorates rheumatoid arthritis via activation of SIRT1-Nrf2 signaling pathway. Biofactors. (2020) 46:441–53. doi: 10.1002/biof.1599 31883358

[67] Deng Y Luo H Shu J Shu H Lu C Zhao N. Pien Tze Huang alleviate the joint inflammation in collagen-induced arthritis mice. Chin Med. (2020) 15:30. doi: 10.1186/s13020-020-00311-3 32256686 PMC7106633

[68] Guo Q Zheng K Fan D Zhao Y Li L Bian Y. Wu-tou decoction in rheumatoid arthritis: integrating network pharmacology and *in vivo* pharmacological evaluation. Front Pharmacol. (2017) 8:230. doi: 10.3389/fphar.2017.00230 28515692 PMC5414545

