# Supplementary material for: Regulating the lncRNA DSCR9/RPLP2/PI3K/AKT axis: an important mechanism of Xinfeng capsules in improving rheumatoid arthritis
Source: Front Immunol. 2024 Sep 23;15:1465442. doi: 10.3389/fimmu.2024.1465442 (PMC11456487; doi:10.3389/fimmu.2024.1465442)
Supplement: Supplementary file 1 [file Table1.docx]

Supplementary Table 1. Correlation Matrix

| Parameter | LncDSCR9 | PI3K | AKT | IL.4 | IL.10 | IL.6 | IL.8 | VEGF | PAF | SAS | SDS | PF | RP | BP | GH | VT | SF | RE | MH | VAS | PLT | FBG | DD | ESR | Hs.CRP | RF | CCP |
| --- | --- | --- | --- | --- | --- | --- | --- | --- | --- | --- | --- | --- | --- | --- | --- | --- | --- | --- | --- | --- | --- | --- | --- | --- | --- | --- | --- |
| LncDSCR9 | 1 | 0.074381753 | -0.2 | 0.002737095 | -0.14977191 | -0.541416567 | -0.024635037 | -0.250036014 | -0.002929172 | -0.964109463 | -0.635291031 | 0.67455113 | 0.365772968 | 0.385258043 | 0.39390499 | 0.585024052 | 0.52019122 | 0.4102947 | 0.481463243 | -0.317147295 | -0.121073865 | -0.123198179 | -0.1211772 | -0.095925075 | -0.021560624 | -0.042016807 | 0.25073302 |
| PI3K | 0.074381753 | 1 | 0.020408163 | 0.16907563 | -0.18665066 | 0.01032413 | -0.209085671 | 0.20547419 | -0.111068427 | -0.029225651 | 0.000961979 | 0.052150171 | -0.095530091 | 0.171318759 | 0.234505451 | 0.10512831 | 0.2754994 | -0.03750972 | 0.156739763 | -0.024635235 | 0.176303911 | 0.096675014 | 0.07139941 | 0.14069011 | 0.005714286 | -0.139495798 | -0.178209323 |
| AKT | -0.2 | 0.020408163 | 1 | -0.150828331 | -0.2240096 | 0.228715486 | 0.051815213 | -0.099255702 | -0.180312125 | 0.196779633 | 0.11120479 | -0.16167539 | -0.074584991 | -0.031104124 | -0.05206371 | -0.001257284 | -0.10804765 | 0.01592879 | -0.000965146 | 0.163766615 | -0.042935357 | 0.153132838 | 0.18637841 | 0.252578656 | 0.027515006 | -0.090516207 | 0.006776568 |
| IL.4 | 0.002737095 | 0.16907563 | -0.150828331 | 1 | -0.07178872 | -0.159183674 | -0.070159432 | -0.063817527 | 0.071020408 | -0.001492579 | -0.029821354 | 0.217423824 | 0.163984808 | 0.103942896 | 0.164163538 | 0.010396774 | -0.01650933 | -0.04239112 | -0.04135652 | -0.067985222 | 0.029920277 | 0.176676952 | 0.03430631 | 0.185935972 | 0.071692677 | -0.158415366 | -0.02191571 |
| IL.10 | -0.149771909 | -0.18665066 | -0.224009604 | -0.071788715 | 1 | 0.065930372 | 0.11390703 | 0.235726291 | -0.100504202 | 0.09759538 | 0.158149372 | -0.05865662 | 0.170115081 | -0.065800971 | -0.012541958 | 0.010396774 | -0.15040194 | -0.13745001 | 0.029533478 | 0.190559315 | -0.040486025 | -0.036709598 | 0.03670872 | -0.010674369 | -0.09454982 | -0.024633854 | -0.11342341 |
| IL.6 | -0.541416567 | 0.01032413 | 0.228715486 | -0.159183673 | 0.06593037 | 1 | 0.018968498 | 0.169747899 | -0.008691477 | 0.531839449 | 0.248623512 | -0.340947765 | -0.286590272 | 0.003888016 | -0.112537338 | -0.188350858 | -0.24778732 | -0.22981128 | -0.138884556 | 0.349258396 | -0.131255404 | -0.055400741 | -0.13583186 | -0.149729666 | -0.193469388 | 0.000336135 | -0.053779998 |
| IL.8 | -0.024635037 | -0.209085671 | 0.051815213 | -0.070159432 | 0.11390703 | 0.018968498 | 1 | 0.02823665 | -0.028572801 | 0.021282304 | 0.083407598 | -0.144208557 | 0.1450901 | -0.290753856 | -0.320199902 | -0.133568691 | -0.18426473 | 0.01284641 | -0.150763095 | 0.037632004 | 0.036957879 | -0.122146962 | -0.03738318 | -0.006034674 | 0.017575874 | 0.038513254 | 0.006800925 |
| VEGF | -0.250036014 | 0.20547419 | -0.099255702 | -0.063817527 | 0.23572629 | 0.169747899 | 0.02823665 | 1 | -0.045378151 | 0.206601763 | 0.237705048 | -0.327885577 | -0.242145791 | -0.131749589 | 0.003743143 | -0.075195272 | -0.01405258 | -0.27592769 | -0.015394084 | 0.269883769 | -0.192344637 | -0.165769781 | 0.11493095 | -0.199495306 | -0.066602641 | -0.080912365 | -0.117268271 |
| PAF | -0.002929172 | -0.111068427 | -0.180312125 | 0.071020408 | -0.1005042 | -0.008691477 | -0.028572801 | -0.045378151 | 1 | 0.018151681 | -0.138813592 | 0.098927594 | 0.042911912 | -0.26763327 | -0.075154525 | -0.100534386 | 0.03891484 | 0.14130375 | 0.057329692 | 0.298583065 | 0.133560658 | -0.029790511 | 0.11051053 | -0.090491725 | 0.095894358 | 0.390828331 | -0.009852457 |
| SAS | -0.964109463 | -0.029225651 | 0.196779633 | -0.001492579 | 0.09759538 | 0.531839449 | 0.021282304 | 0.206601763 | 0.018151681 | 1 | 0.635568206 | -0.590631585 | -0.321932176 | -0.34197523 | -0.403683862 | -0.558297579 | -0.48975705 | -0.33185752 | -0.488775639 | 0.282503721 | 0.189608869 | 0.161780986 | 0.16452316 | 0.10346167 | 0.059895734 | 0.08170664 | -0.251548492 |
| SDS | -0.635291031 | 0.000961979 | 0.11120479 | -0.029821354 | 0.15814937 | 0.248623512 | 0.083407598 | 0.237705048 | -0.138813591 | 0.635568206 | 1 | -0.381382818 | -0.217730275 | -0.279613279 | -0.29198743 | -0.416440539 | -0.44910062 | -0.20915519 | -0.415532987 | 0.143433258 | -0.020565267 | 0.044254626 | 0.23570617 | 0.061792615 | -0.050984895 | -0.153387576 | -0.260320217 |
| PF | 0.67455113 | 0.052150171 | -0.16167539 | 0.217423824 | -0.05865662 | -0.340947765 | -0.144208557 | -0.327885577 | 0.098927594 | -0.590631585 | -0.381382818 | 1 | 0.461462419 | 0.443936974 | 0.332883038 | 0.620054432 | 0.38747863 | 0.37811117 | 0.469647909 | -0.184663061 | -0.073701078 | 0.005992632 | -0.05943167 | -0.135138089 | -0.06792338 | -0.047812539 | 0.051282628 |
| RP | 0.365772968 | -0.095530091 | -0.074584991 | 0.163984808 | 0.17011508 | -0.286590272 | 0.1450901 | -0.242145791 | 0.042911912 | -0.321932176 | -0.217730275 | 0.461462419 | 1 | -0.043980866 | -0.027926917 | 0.249251348 | 0.05854526 | 0.64708983 | 0.283904247 | -0.089941209 | 0.003831973 | -0.064919263 | -0.18964148 | -0.071102953 | -0.149169981 | -0.035759927 | 0.051385472 |
| BP | 0.385258043 | 0.171318759 | -0.031104124 | 0.103942896 | -0.06580097 | 0.003888016 | -0.290753856 | -0.131749589 | -0.26763327 | -0.34197523 | -0.27961328 | 0.443936974 | -0.043980866 | 1 | 0.5398592 | 0.629656406 | 0.47521151 | 0.13178955 | 0.423619983 | -0.155324502 | 0.132211575 | 0.203755894 | 0.07637892 | 0.129509168 | 0.097643326 | -0.178651852 | 0.197253583 |
| GH | 0.39390499 | 0.234505451 | -0.05206371 | 0.164163538 | -0.01254196 | -0.112537338 | -0.320199902 | 0.003743143 | -0.075154525 | -0.403683862 | -0.29198743 | 0.332883038 | -0.027926917 | 0.5398592 | 1 | 0.526405527 | 0.53335554 | 0.07711633 | 0.464668069 | 0.010260233 | -0.141457695 | 0.158939747 | 0.26086408 | 0.029522335 | 0.152545213 | -0.018910162 | 0.121976364 |
| VT | 0.585024052 | 0.10512831 | -0.001257284 | 0.010396774 | 0.01039677 | -0.188350858 | -0.133568691 | -0.075195272 | -0.100534386 | -0.558297579 | -0.416440539 | 0.620054432 | 0.249251348 | 0.629656406 | 0.526405527 | 1 | 0.68144729 | 0.4056787 | 0.738405171 | -0.064699356 | -0.096486291 | 0.139282853 | 0.07045022 | 0.034863172 | 0.018665836 | -0.043569736 | 0.14880254 |
| SF | 0.520191219 | 0.275499401 | -0.108047652 | -0.016509327 | -0.15040194 | -0.247787316 | -0.184264735 | -0.014052582 | 0.038914843 | -0.48975705 | -0.449100616 | 0.387478629 | 0.058545257 | 0.475211514 | 0.533355541 | 0.68144729 | 1 | 0.32860648 | 0.655327344 | -0.085300324 | -0.049952826 | 0.251211655 | 0.14776367 | 0.13438957 | 0.11674453 | 0.162538259 | 0.318202546 |
| RE | 0.410294705 | -0.037509723 | 0.015928786 | -0.042391125 | -0.13745001 | -0.22981128 | 0.012846412 | -0.275927685 | 0.141303749 | -0.331857522 | -0.209155191 | 0.378111172 | 0.647089835 | 0.131789553 | 0.077116329 | 0.4056787 | 0.32860648 | 1 | 0.466097233 | 0.14120074 | -0.023575431 | 0.191779023 | 0.03528289 | 0.020065956 | -0.06217365 | -0.032499862 | 0.191182297 |
| MH | 0.481463243 | 0.156739763 | -0.000965146 | -0.04135652 | 0.02953348 | -0.138884556 | -0.150763095 | -0.015394084 | 0.057329692 | -0.488775639 | -0.415532987 | 0.469647909 | 0.283904247 | 0.423619983 | 0.464668069 | 0.738405171 | 0.65532734 | 0.46609723 | 1 | 0.111458685 | -0.146650983 | 0.036505327 | 0.07023244 | -0.092124306 | -0.064085716 | 0.042418181 | 0.034654591 |
| VAS | -0.317147295 | -0.024635235 | 0.163766615 | -0.067985222 | 0.19055931 | 0.349258396 | 0.037632004 | 0.269883769 | 0.298583065 | 0.282503721 | 0.143433258 | -0.184663061 | -0.089941209 | -0.155324502 | 0.010260233 | -0.064699356 | -0.08530032 | 0.14120074 | 0.111458685 | 1 | -0.237280029 | 0.039511292 | 0.12458058 | -0.1142206 | -0.072551019 | -0.005619443 | -0.083862421 |
| PLT | -0.121073865 | 0.176303911 | -0.042935357 | 0.029920277 | -0.04048602 | -0.131255404 | 0.036957879 | -0.192344637 | 0.133560658 | 0.189608869 | -0.020565267 | -0.073701078 | 0.003831973 | 0.132211575 | -0.141457695 | -0.096486291 | -0.04995283 | -0.02357543 | -0.146650983 | -0.237280029 | 1 | 0.338627556 | 0.34489059 | 0.365938363 | 0.397848434 | 0.081644415 | -0.094164608 |
| FBG | -0.123198179 | 0.096675014 | 0.153132838 | 0.176676952 | -0.0367096 | -0.055400741 | -0.122146962 | -0.165769781 | -0.029790511 | 0.161780986 | 0.044254626 | 0.005992632 | -0.064919263 | 0.203755894 | 0.158939747 | 0.139282853 | 0.25121165 | 0.19177902 | 0.036505327 | 0.039511292 | 0.338627556 | 1 | 0.62633717 | 0.806514666 | 0.727417017 | 0.058283694 | 0.027075119 |
| DD | -0.121177199 | 0.071399412 | 0.186378412 | 0.034306312 | 0.03670872 | -0.135831856 | -0.037383183 | 0.114930952 | 0.11051053 | 0.164523165 | 0.235706167 | -0.059431673 | -0.189641478 | 0.076378923 | 0.260864077 | 0.07045022 | 0.14776367 | 0.03528289 | 0.070232435 | 0.124580577 | 0.344890591 | 0.626337172 | 1 | 0.535554626 | 0.639183298 | 0.083123138 | -0.179566719 |
| ESR | -0.095925075 | 0.14069011 | 0.252578656 | 0.185935972 | -0.01067437 | -0.149729666 | -0.006034674 | -0.199495306 | -0.090491725 | 0.10346167 | 0.061792615 | -0.135138089 | -0.071102953 | 0.129509168 | 0.029522335 | 0.034863172 | 0.13438957 | 0.02006596 | -0.092124306 | -0.1142206 | 0.365938363 | 0.806514666 | 0.53555463 | 1 | 0.682390307 | 0.040437588 | 0.033374243 |
| Hs.CRP | -0.021560624 | 0.005714286 | 0.027515006 | 0.071692677 | -0.09454982 | -0.193469388 | 0.017575874 | -0.066602641 | 0.095894358 | 0.059895734 | -0.050984895 | -0.06792338 | -0.149169981 | 0.097643326 | 0.152545213 | 0.018665836 | 0.11674453 | -0.06217365 | -0.064085716 | -0.072551019 | 0.397848434 | 0.727417017 | 0.6391833 | 0.682390307 | 1 | 0.237262905 | 0.078242928 |
| RF | -0.042016807 | -0.139495798 | -0.090516207 | -0.158415366 | -0.02463385 | 0.000336135 | 0.038513254 | -0.080912365 | 0.390828331 | 0.08170664 | -0.153387576 | -0.047812539 | -0.035759927 | -0.178651852 | -0.018910162 | -0.043569736 | 0.16253826 | -0.03249986 | 0.042418181 | -0.005619443 | 0.081644415 | 0.058283694 | 0.08312314 | 0.040437588 | 0.237262905 | 1 | 0.21497581 |
| CCP | 0.25073302 | -0.178209323 | 0.006776568 | -0.02191571 | -0.11342341 | -0.053779998 | 0.006800925 | -0.117268271 | -0.009852457 | -0.251548492 | -0.260320217 | 0.051282628 | 0.051385472 | 0.197253583 | 0.121976364 | 0.14880254 | 0.31820255 | 0.1911823 | 0.034654591 | -0.083862421 | -0.094164608 | 0.027075119 | -0.17956672 | 0.033374243 | 0.078242928 | 0.21497581 | 1 |

Supplementary Table 2. Correlation analysis results

| Parameter1 | Parameter2 | rho | CI | CI_low | CI_high | S | p |
| --- | --- | --- | --- | --- | --- | --- | --- |
| LncDSCR9 | PI3K | 0.074381753 | 0.95 | -0.216348893 | 0.352995293 | 19,276.00 | 0.608 |
| LncDSCR9 | AKT | -0.2 | 0.95 | -0.459813263 | 0.091353937 | 24,990.00 | 0.164 |
| LncDSCR9 | IL.4 | 0.002737095 | 0.95 | -0.283611203 | 0.288637234 | 20,768.00 | 0.985 |
| LncDSCR9 | IL.10 | -0.149771909 | 0.95 | -0.417985869 | 0.142459175 | 23,944.00 | 0.299 |
| LncDSCR9 | IL.6 | -0.541416567 | 0.95 | -0.716540921 | -0.302087854 | 32,100.00 | <0.001 |
| LncDSCR9 | IL.8 | -0.024635037 | 0.95 | -0.308586083 | 0.263347414 | 21,338.03 | 0.865 |
| LncDSCR9 | VEGF | -0.250036014 | 0.95 | -0.500365151 | 0.038871081 | 26,032.00 | 0.08 |
| LncDSCR9 | PAF | -0.002929172 | 0.95 | -0.2888133 | 0.283434565 | 20,886.00 | 0.984 |
| LncDSCR9 | SAS | -0.964109463 | 0.95 | -0.979918349 | -0.93625602 | 40,902.58 | <0.001 |
| LncDSCR9 | SDS | -0.635291031 | 0.95 | -0.779690267 | -0.426733676 | 34,054.94 | <0.001 |
| LncDSCR9 | PF | 0.67455113 | 0.95 | 0.481323654 | 0.80525724 | 6,777.47 | <0.001 |
| LncDSCR9 | RP | 0.365772968 | 0.95 | 0.088956748 | 0.590137048 | 13,207.78 | 0.009 |
| LncDSCR9 | BP | 0.385258043 | 0.95 | 0.111413203 | 0.604724038 | 12,802.00 | 0.006 |
| LncDSCR9 | GH | 0.39390499 | 0.95 | 0.121469173 | 0.61115052 | 12,621.93 | 0.005 |
| LncDSCR9 | VT | 0.585024052 | 0.95 | 0.358989344 | 0.746237089 | 8,641.87 | <0.001 |
| LncDSCR9 | SF | 0.520191219 | 0.95 | 0.274995445 | 0.701853325 | 9,992.02 | <0.001 |
| LncDSCR9 | RE | 0.410294705 | 0.95 | 0.140684301 | 0.623253398 | 12,280.61 | 0.003 |
| LncDSCR9 | MH | 0.481463243 | 0.95 | 0.22654584 | 0.674650138 | 10,798.53 | <0.001 |
| LncDSCR9 | VAS | -0.317147295 | 0.95 | -0.553084317 | -0.03411703 | 27,429.59 | 0.025 |
| LncDSCR9 | PLT | -0.121073865 | 0.95 | -0.39356598 | 0.170975317 | 23,346.36 | 0.402 |
| LncDSCR9 | FBG | -0.123198179 | 0.95 | -0.395386888 | 0.168881104 | 23,390.60 | 0.394 |
| LncDSCR9 | DD | -0.121177199 | 0.95 | -0.393654605 | 0.170873508 | 23,348.52 | 0.402 |
| LncDSCR9 | ESR | -0.095925075 | 0.95 | -0.371845343 | 0.195568826 | 22,822.64 | 0.508 |
| LncDSCR9 | Hs.CRP | -0.021560624 | 0.95 | -0.30580031 | 0.266207814 | 21,274.00 | 0.882 |
| LncDSCR9 | RF | -0.042016807 | 0.95 | -0.324244886 | 0.2470798 | 21,700.00 | 0.772 |
| LncDSCR9 | CCP | 0.25073302 | 0.95 | -0.038128558 | 0.500922392 | 15,603.49 | 0.079 |
| PI3K | AKT | 0.020408163 | 0.95 | -0.267278746 | 0.304754792 | 20,400.00 | 0.888 |
| PI3K | IL.4 | 0.16907563 | 0.95 | -0.12300097 | 0.434196698 | 17,304.00 | 0.24 |
| PI3K | IL.10 | -0.18665066 | 0.95 | -0.448807969 | 0.105087808 | 24,712.00 | 0.194 |
| PI3K | IL.6 | 0.01032413 | 0.95 | -0.276619191 | 0.29557718 | 20,610.00 | 0.943 |
| PI3K | IL.8 | -0.209085671 | 0.95 | -0.467258189 | 0.08194273 | 25,179.21 | 0.145 |
| PI3K | VEGF | 0.20547419 | 0.95 | -0.085689834 | 0.464303269 | 16,546.00 | 0.152 |
| PI3K | PAF | -0.111068427 | 0.95 | -0.384960721 | 0.180803623 | 23,138.00 | 0.443 |
| PI3K | SAS | -0.029225651 | 0.95 | -0.312736664 | 0.25906691 | 21,433.62 | 0.84 |
| PI3K | SDS | 0.000961979 | 0.95 | -0.285242719 | 0.287009167 | 20,804.97 | 0.995 |
| PI3K | PF | 0.052150171 | 0.95 | -0.237520179 | 0.333302968 | 19,738.97 | 0.719 |
| PI3K | RP | -0.095530091 | 0.95 | -0.371501774 | 0.195952186 | 22,814.41 | 0.509 |
| PI3K | BP | 0.171318759 | 0.95 | -0.120725231 | 0.436069356 | 17,257.29 | 0.234 |
| PI3K | GH | 0.234505451 | 0.95 | -0.055333511 | 0.487894799 | 15,941.42 | 0.101 |
| PI3K | VT | 0.10512831 | 0.95 | -0.186611133 | 0.379829246 | 18,635.70 | 0.467 |
| PI3K | SF | 0.275499401 | 0.95 | -0.011536152 | 0.520588824 | 15,087.73 | 0.053 |
| PI3K | RE | -0.037509723 | 0.95 | -0.320199365 | 0.251313691 | 21,606.14 | 0.796 |
| PI3K | MH | 0.156739763 | 0.95 | -0.135461513 | 0.423857082 | 17,560.89 | 0.277 |
| PI3K | VAS | -0.024635235 | 0.95 | -0.308586263 | 0.263347229 | 21,338.03 | 0.865 |
| PI3K | PLT | 0.176303911 | 0.95 | -0.115656591 | 0.440222976 | 17,153.47 | 0.221 |
| PI3K | FBG | 0.096675014 | 0.95 | -0.19484071 | 0.372497454 | 18,811.74 | 0.504 |
| PI3K | DD | 0.071399412 | 0.95 | -0.219204965 | 0.350367849 | 19,338.11 | 0.622 |
| PI3K | ESR | 0.14069011 | 0.95 | -0.151536184 | 0.410299633 | 17,895.13 | 0.33 |
| PI3K | Hs.CRP | 0.005714286 | 0.95 | -0.280871127 | 0.29136409 | 20,706.00 | 0.969 |
| PI3K | RF | -0.139495798 | 0.95 | -0.409285989 | 0.15272621 | 23,730.00 | 0.334 |
| PI3K | CCP | -0.178209323 | 0.95 | -0.441807584 | 0.113715244 | 24,536.21 | 0.216 |
| AKT | IL.4 | -0.150828331 | 0.95 | -0.418877469 | 0.141400109 | 23,966.00 | 0.296 |
| AKT | IL.10 | -0.224009604 | 0.95 | -0.47940812 | 0.066370607 | 25,490.00 | 0.118 |
| AKT | IL.6 | 0.228715486 | 0.95 | -0.061430825 | 0.483219095 | 16,062.00 | 0.11 |
| AKT | IL.8 | 0.051815213 | 0.95 | -0.237837072 | 0.33300438 | 19,745.95 | 0.721 |
| AKT | VEGF | -0.099255702 | 0.95 | -0.374739418 | 0.192332665 | 22,892.00 | 0.493 |
| AKT | PAF | -0.180312125 | 0.95 | -0.443554441 | 0.111570193 | 24,580.00 | 0.21 |
| AKT | SAS | 0.196779633 | 0.95 | -0.094677242 | 0.457165653 | 16,727.06 | 0.171 |
| AKT | SDS | 0.11120479 | 0.95 | -0.180670065 | 0.385078322 | 18,509.16 | 0.442 |
| AKT | PF | -0.16167539 | 0.95 | -0.428002373 | 0.130487072 | 24,191.89 | 0.262 |
| AKT | RP | -0.074584991 | 0.95 | -0.353174186 | 0.216154079 | 22,378.23 | 0.607 |
| AKT | BP | -0.031104124 | 0.95 | -0.314431959 | 0.257312064 | 21,472.74 | 0.83 |
| AKT | GH | -0.05206371 | 0.95 | -0.3332259 | 0.237601983 | 21,909.23 | 0.72 |
| AKT | VT | -0.001257284 | 0.95 | -0.287280124 | 0.284971418 | 20,851.18 | 0.993 |
| AKT | SF | -0.108047652 | 0.95 | -0.382353286 | 0.183759508 | 23,075.09 | 0.455 |
| AKT | RE | 0.015928786 | 0.95 | -0.271434501 | 0.30068456 | 20,493.28 | 0.913 |
| AKT | MH | -0.000965146 | 0.95 | -0.287012073 | 0.28523981 | 20,845.10 | 0.995 |
| AKT | VAS | 0.163766615 | 0.95 | -0.128374954 | 0.42975536 | 17,414.56 | 0.256 |
| AKT | PLT | -0.042935357 | 0.95 | -0.325068105 | 0.24621557 | 21,719.13 | 0.767 |
| AKT | FBG | 0.153132838 | 0.95 | -0.139087511 | 0.420820636 | 17,636.01 | 0.288 |
| AKT | DD | 0.186378412 | 0.95 | -0.105366745 | 0.448582695 | 16,943.67 | 0.195 |
| AKT | ESR | 0.252578656 | 0.95 | -0.036160852 | 0.502396933 | 15,565.05 | 0.077 |
| AKT | Hs.CRP | 0.027515006 | 0.95 | -0.260663321 | 0.311191257 | 20,252.00 | 0.85 |
| AKT | RF | -0.090516207 | 0.95 | -0.367133969 | 0.20081079 | 22,710.00 | 0.532 |
| AKT | CCP | 0.006776568 | 0.95 | -0.279892316 | 0.292335929 | 20,683.88 | 0.963 |
| IL.4 | IL.10 | -0.071788716 | 0.95 | -0.350711075 | 0.218832427 | 22,320.00 | 0.62 |
| IL.4 | IL.6 | -0.159183674 | 0.95 | -0.425911052 | 0.133000223 | 24,140.00 | 0.27 |
| IL.4 | IL.8 | -0.070159432 | 0.95 | -0.349274131 | 0.220390983 | 22,286.07 | 0.628 |
| IL.4 | VEGF | -0.063817527 | 0.95 | -0.343668375 | 0.226443487 | 22,154.00 | 0.66 |
| IL.4 | PAF | 0.071020408 | 0.95 | -0.219567566 | 0.350033632 | 19,346.00 | 0.624 |
| IL.4 | SAS | -0.001492579 | 0.95 | -0.287495985 | 0.284755217 | 20,856.08 | 0.992 |
| IL.4 | SDS | -0.029821354 | 0.95 | -0.313274472 | 0.258510618 | 21,446.03 | 0.837 |
| IL.4 | PF | 0.217423824 | 0.95 | -0.073259909 | 0.474058522 | 16,297.15 | 0.129 |
| IL.4 | RP | 0.163984808 | 0.95 | -0.128154429 | 0.429938147 | 17,410.02 | 0.255 |
| IL.4 | BP | 0.103942896 | 0.95 | -0.187767648 | 0.378803178 | 18,660.39 | 0.473 |
| IL.4 | GH | 0.164163538 | 0.95 | -0.127973766 | 0.430087858 | 17,406.29 | 0.255 |
| IL.4 | VT | 0.010396774 | 0.95 | -0.276552097 | 0.295643483 | 20,608.49 | 0.943 |
| IL.4 | SF | -0.016509327 | 0.95 | -0.30121266 | 0.270896506 | 21,168.81 | 0.909 |
| IL.4 | RE | -0.042391125 | 0.95 | -0.324580407 | 0.246727673 | 21,707.80 | 0.77 |
| IL.4 | MH | -0.04135652 | 0.95 | -0.323652863 | 0.247700755 | 21,686.25 | 0.776 |
| IL.4 | VAS | -0.067985222 | 0.95 | -0.347354545 | 0.2224685 | 22,240.79 | 0.639 |
| IL.4 | PLT | 0.029920277 | 0.95 | -0.258418221 | 0.313363764 | 20,201.91 | 0.837 |
| IL.4 | FBG | 0.176676952 | 0.95 | -0.115276691 | 0.44053334 | 17,145.70 | 0.22 |
| IL.4 | DD | 0.034306313 | 0.95 | -0.254316225 | 0.317317729 | 20,110.57 | 0.813 |
| IL.4 | ESR | 0.185935972 | 0.95 | -0.105819958 | 0.448216524 | 16,952.88 | 0.196 |
| IL.4 | Hs.CRP | 0.071692677 | 0.95 | -0.218924337 | 0.35062641 | 19,332.00 | 0.621 |
| IL.4 | RF | -0.158415366 | 0.95 | -0.425265629 | 0.133774383 | 24,124.00 | 0.272 |
| IL.4 | CCP | -0.02191571 | 0.95 | -0.306122307 | 0.265877705 | 21,281.40 | 0.88 |
| IL.10 | IL.6 | 0.065930372 | 0.95 | -0.224429544 | 0.345538186 | 19,452.00 | 0.649 |
| IL.10 | IL.8 | 0.113907031 | 0.95 | -0.178021184 | 0.387406934 | 18,452.89 | 0.431 |
| IL.10 | VEGF | 0.235726291 | 0.95 | -0.054045101 | 0.488878839 | 15,916.00 | 0.099 |
| IL.10 | PAF | -0.100504202 | 0.95 | -0.375822893 | 0.191117941 | 22,918.00 | 0.487 |
| IL.10 | SAS | 0.09759538 | 0.95 | -0.193946689 | 0.373297388 | 18,792.58 | 0.5 |
| IL.10 | SDS | 0.158149372 | 0.95 | -0.13404232 | 0.425042115 | 17,531.54 | 0.273 |
| IL.10 | PF | -0.05865662 | 0.95 | -0.339091763 | 0.231352399 | 22,046.52 | 0.686 |
| IL.10 | RP | 0.170115081 | 0.95 | -0.121946789 | 0.43506476 | 17,282.35 | 0.238 |
| IL.10 | BP | -0.065800971 | 0.95 | -0.345423733 | 0.224552959 | 22,195.31 | 0.65 |
| IL.10 | GH | -0.012541958 | 0.95 | -0.297600182 | 0.274569544 | 21,086.19 | 0.931 |
| IL.10 | VT | 0.010396774 | 0.95 | -0.276552097 | 0.295643483 | 20,608.49 | 0.943 |
| IL.10 | SF | -0.150401938 | 0.95 | -0.418517663 | 0.14182765 | 23,957.12 | 0.297 |
| IL.10 | RE | -0.137450011 | 0.95 | -0.407548125 | 0.15476269 | 23,687.40 | 0.341 |
| IL.10 | MH | 0.029533478 | 0.95 | -0.258779473 | 0.313014596 | 20,209.97 | 0.839 |
| IL.10 | VAS | 0.190559315 | 0.95 | -0.101078052 | 0.452038547 | 16,856.60 | 0.185 |
| IL.10 | PLT | -0.040486025 | 0.95 | -0.322872025 | 0.248519034 | 21,668.12 | 0.78 |
| IL.10 | FBG | -0.036709598 | 0.95 | -0.3194801 | 0.252064164 | 21,589.48 | 0.8 |
| IL.10 | DD | 0.036708715 | 0.95 | -0.252064991 | 0.319479307 | 20,060.54 | 0.8 |
| IL.10 | ESR | -0.010674369 | 0.95 | -0.295896823 | 0.276295684 | 21,047.29 | 0.941 |
| IL.10 | Hs.CRP | -0.09454982 | 0.95 | -0.370648778 | 0.196903225 | 22,794.00 | 0.514 |
| IL.10 | RF | -0.024633854 | 0.95 | -0.308585012 | 0.263348516 | 21,338.00 | 0.865 |
| IL.10 | CCP | -0.11342341 | 0.95 | -0.386990437 | 0.178495566 | 23,187.04 | 0.433 |
| IL.6 | IL.8 | 0.018968498 | 0.95 | -0.268615568 | 0.303447757 | 20,429.98 | 0.896 |
| IL.6 | VEGF | 0.169747899 | 0.95 | -0.12231925 | 0.434758177 | 17,290.00 | 0.239 |
| IL.6 | PAF | -0.008691477 | 0.95 | -0.294086312 | 0.278126371 | 21,006.00 | 0.952 |
| IL.6 | SAS | 0.531839449 | 0.95 | 0.289815397 | 0.709932882 | 9,749.44 | <0.001 |
| IL.6 | SDS | 0.248623512 | 0.95 | -0.040374848 | 0.499235252 | 15,647.42 | 0.082 |
| IL.6 | PF | -0.340947765 | 0.95 | -0.571337632 | -0.060747772 | 27,925.24 | 0.015 |
| IL.6 | RP | -0.286590272 | 0.95 | -0.529312328 | -0.000505541 | 26,793.24 | 0.044 |
| IL.6 | BP | 0.003888016 | 0.95 | -0.2825525 | 0.28969193 | 20,744.03 | 0.979 |
| IL.6 | GH | -0.112537338 | 0.95 | -0.38622706 | 0.179364359 | 23,168.59 | 0.437 |
| IL.6 | VT | -0.188350858 | 0.95 | -0.450214058 | 0.103344798 | 24,747.41 | 0.19 |
| IL.6 | SF | -0.247787316 | 0.95 | -0.498565954 | 0.041264456 | 25,985.17 | 0.083 |
| IL.6 | RE | -0.22981128 | 0.95 | -0.484105122 | 0.060278522 | 25,610.82 | 0.108 |
| IL.6 | MH | -0.138884556 | 0.95 | -0.408766953 | 0.153334932 | 23,717.27 | 0.336 |
| IL.6 | VAS | 0.349258396 | 0.95 | 0.070141598 | 0.577658072 | 13,551.69 | 0.013 |
| IL.6 | PLT | -0.131255404 | 0.95 | -0.402273948 | 0.160914005 | 23,558.39 | 0.364 |
| IL.6 | FBG | -0.055400741 | 0.95 | -0.336197656 | 0.234441722 | 21,978.72 | 0.702 |
| IL.6 | DD | -0.135831856 | 0.95 | -0.406172142 | 0.156371725 | 23,653.70 | 0.347 |
| IL.6 | ESR | -0.149729666 | 0.95 | -0.417950206 | 0.14250151 | 23,943.12 | 0.299 |
| IL.6 | Hs.CRP | -0.193469388 | 0.95 | -0.454439331 | 0.098086542 | 24,854.00 | 0.178 |
| IL.6 | RF | 0.000336135 | 0.95 | -0.285817541 | 0.286434772 | 20,818.00 | 0.998 |
| IL.6 | CCP | -0.053779998 | 0.95 | -0.334755019 | 0.235977376 | 21,944.97 | 0.711 |
| IL.8 | VEGF | 0.02823665 | 0.95 | -0.25999006 | 0.311843377 | 20,236.97 | 0.846 |
| IL.8 | PAF | -0.028572801 | 0.95 | -0.312147052 | 0.259676351 | 21,420.03 | 0.844 |
| IL.8 | SAS | 0.021282304 | 0.95 | -0.26646651 | 0.305547879 | 20,381.80 | 0.883 |
| IL.8 | SDS | 0.083407598 | 0.95 | -0.207674767 | 0.360920384 | 19,088.04 | 0.565 |
| IL.8 | PF | -0.144208557 | 0.95 | -0.413281967 | 0.148025432 | 23,828.14 | 0.318 |
| IL.8 | RP | 0.1450901 | 0.95 | -0.147144661 | 0.414028284 | 17,803.50 | 0.315 |
| IL.8 | BP | -0.290753856 | 0.95 | -0.532573991 | -0.00504759 | 26,879.95 | 0.041 |
| IL.8 | GH | -0.320199902 | 0.95 | -0.555438188 | -0.037510321 | 27,493.16 | 0.023 |
| IL.8 | VT | -0.133568691 | 0.95 | -0.404245621 | 0.158619537 | 23,606.57 | 0.355 |
| IL.8 | SF | -0.184264735 | 0.95 | -0.446832582 | 0.107530794 | 24,662.31 | 0.2 |
| IL.8 | RE | 0.012846412 | 0.95 | -0.274287973 | 0.297877691 | 20,557.47 | 0.929 |
| IL.8 | MH | -0.150763095 | 0.95 | -0.418822426 | 0.141465528 | 23,964.64 | 0.296 |
| IL.8 | VAS | 0.037632004 | 0.95 | -0.251198968 | 0.320309259 | 20,041.31 | 0.795 |
| IL.8 | PLT | 0.036957879 | 0.95 | -0.251831327 | 0.319703325 | 20,055.35 | 0.799 |
| IL.8 | FBG | -0.122146962 | 0.95 | -0.394486079 | 0.169917756 | 23,368.71 | 0.398 |
| IL.8 | DD | -0.037383183 | 0.95 | -0.320085635 | 0.251432401 | 21,603.51 | 0.797 |
| IL.8 | ESR | -0.006034674 | 0.95 | -0.291657262 | 0.280575977 | 20,950.67 | 0.967 |
| IL.8 | Hs.CRP | 0.017575874 | 0.95 | -0.269907656 | 0.30218241 | 20,458.98 | 0.904 |
| IL.8 | RF | 0.038513254 | 0.95 | -0.250371944 | 0.321101019 | 20,022.96 | 0.791 |
| IL.8 | CCP | 0.006800925 | 0.95 | -0.279869866 | 0.292358205 | 20,683.37 | 0.963 |
| VEGF | PAF | -0.045378151 | 0.95 | -0.327255295 | 0.243914997 | 21,770.00 | 0.754 |
| VEGF | SAS | 0.206601763 | 0.95 | -0.0845208 | 0.465226471 | 16,522.52 | 0.15 |
| VEGF | SDS | 0.237705048 | 0.95 | -0.051954771 | 0.49047242 | 15,874.79 | 0.096 |
| VEGF | PF | -0.327885577 | 0.95 | -0.561347976 | -0.046082716 | 27,653.22 | 0.02 |
| VEGF | RP | -0.242145791 | 0.95 | -0.494042605 | 0.047254379 | 25,867.69 | 0.09 |
| VEGF | BP | -0.131749589 | 0.95 | -0.402695365 | 0.160424106 | 23,568.69 | 0.362 |
| VEGF | GH | 0.003743143 | 0.95 | -0.282685803 | 0.289559208 | 20,747.05 | 0.979 |
| VEGF | VT | -0.075195272 | 0.95 | -0.353711242 | 0.215568953 | 22,390.94 | 0.604 |
| VEGF | SF | -0.014052582 | 0.95 | -0.29897664 | 0.273171977 | 21,117.65 | 0.923 |
| VEGF | RE | -0.275927685 | 0.95 | -0.520926642 | 0.011072692 | 26,571.19 | 0.052 |
| VEGF | MH | -0.015394084 | 0.95 | -0.300198003 | 0.271929858 | 21,145.58 | 0.915 |
| VEGF | VAS | 0.269883769 | 0.95 | -0.01760163 | 0.516152258 | 15,204.67 | 0.058 |
| VEGF | PLT | -0.192344638 | 0.95 | -0.453511872 | 0.099243393 | 24,830.58 | 0.181 |
| VEGF | FBG | -0.165769781 | 0.95 | -0.431432652 | 0.126349288 | 24,277.16 | 0.25 |
| VEGF | DD | 0.114930952 | 0.95 | -0.177016375 | 0.38828837 | 18,431.56 | 0.427 |
| VEGF | ESR | -0.199495306 | 0.95 | -0.459398636 | 0.091875193 | 24,979.49 | 0.165 |
| VEGF | Hs.CRP | -0.066602641 | 0.95 | -0.34613266 | 0.223788224 | 22,212.00 | 0.646 |
| VEGF | RF | -0.080912365 | 0.95 | -0.35873346 | 0.210077353 | 22,510.00 | 0.576 |
| VEGF | CCP | -0.117268271 | 0.95 | -0.390298565 | 0.1747204 | 23,267.11 | 0.417 |
| PAF | SAS | 0.018151681 | 0.95 | -0.269373543 | 0.302705714 | 20,446.99 | 0.9 |
| PAF | SDS | -0.138813592 | 0.95 | -0.408706683 | 0.153405589 | 23,715.79 | 0.336 |
| PAF | PF | 0.098927594 | 0.95 | -0.19265175 | 0.374454553 | 18,764.83 | 0.494 |
| PAF | RP | 0.042911912 | 0.95 | -0.246237634 | 0.325047098 | 19,931.36 | 0.767 |
| PAF | BP | -0.26763327 | 0.95 | -0.51437056 | 0.020026482 | 26,398.46 | 0.06 |
| PAF | GH | -0.075154525 | 0.95 | -0.353675389 | 0.215608027 | 22,390.09 | 0.604 |
| PAF | VT | -0.100534386 | 0.95 | -0.375849078 | 0.191088562 | 22,918.63 | 0.487 |
| PAF | SF | 0.038914843 | 0.95 | -0.249994925 | 0.321461696 | 20,014.60 | 0.788 |
| PAF | RE | 0.141303749 | 0.95 | -0.150924417 | 0.410820187 | 17,882.35 | 0.328 |
| PAF | MH | 0.057329692 | 0.95 | -0.232612154 | 0.337912915 | 19,631.11 | 0.693 |
| PAF | VAS | 0.298583065 | 0.95 | 0.013620512 | 0.538687838 | 14,607.01 | 0.035 |
| PAF | PLT | 0.133560658 | 0.95 | -0.15862751 | 0.404238778 | 18,043.60 | 0.355 |
| PAF | FBG | -0.029790511 | 0.95 | -0.313246632 | 0.258539425 | 21,445.39 | 0.837 |
| PAF | DD | 0.11051053 | 0.95 | -0.181349933 | 0.384479492 | 18,523.62 | 0.445 |
| PAF | ESR | -0.090491725 | 0.95 | -0.367112612 | 0.200834478 | 22,709.49 | 0.532 |
| PAF | Hs.CRP | 0.095894358 | 0.95 | -0.195598642 | 0.371818627 | 18,828.00 | 0.508 |
| PAF | RF | 0.390828331 | 0.95 | 0.117884751 | 0.6088672 | 12,686.00 | 0.005 |
| PAF | CCP | -0.009852457 | 0.95 | -0.295146612 | 0.277054759 | 21,030.18 | 0.946 |
| SAS | SDS | 0.635568206 | 0.95 | 0.427113825 | 0.779872472 | 7,589.29 | <0.001 |
| SAS | PF | -0.590631585 | 0.95 | -0.750009751 | -0.366430356 | 33,124.90 | <0.001 |
| SAS | RP | -0.321932176 | 0.95 | -0.556772272 | -0.039438827 | 27,529.24 | 0.023 |
| SAS | BP | -0.34197523 | 0.95 | -0.572120528 | -0.061906473 | 27,946.63 | 0.015 |
| SAS | GH | -0.403683862 | 0.95 | -0.618383909 | -0.132909302 | 29,231.72 | 0.004 |
| SAS | VT | -0.558297579 | 0.95 | -0.728112487 | -0.323914675 | 32,451.55 | <0.001 |
| SAS | SF | -0.48975705 | 0.95 | -0.680520344 | -0.23681651 | 31,024.19 | <0.001 |
| SAS | RE | -0.331857522 | 0.95 | -0.564392843 | -0.050529246 | 27,735.93 | 0.019 |
| SAS | MH | -0.488775639 | 0.95 | -0.679826994 | -0.235598219 | 31,003.75 | <0.001 |
| SAS | VAS | 0.282503721 | 0.95 | -0.003941025 | 0.526104019 | 14,941.86 | 0.047 |
| SAS | PLT | 0.189608869 | 0.95 | -0.102053955 | 0.451253616 | 16,876.40 | 0.187 |
| SAS | FBG | 0.161780986 | 0.95 | -0.130380484 | 0.428090939 | 17,455.91 | 0.262 |
| SAS | DD | 0.164523165 | 0.95 | -0.127610193 | 0.430389051 | 17,398.81 | 0.254 |
| SAS | ESR | 0.10346167 | 0.95 | -0.188236911 | 0.378386447 | 18,670.41 | 0.475 |
| SAS | Hs.CRP | 0.059895734 | 0.95 | -0.230175134 | 0.340191802 | 19,577.67 | 0.679 |
| SAS | RF | 0.08170664 | 0.95 | -0.20931295 | 0.359429926 | 19,123.46 | 0.573 |
| SAS | CCP | -0.251548492 | 0.95 | -0.501574079 | 0.037259427 | 26,063.50 | 0.078 |
| SDS | PF | -0.381382818 | 0.95 | -0.601834636 | -0.106924634 | 28,767.30 | 0.006 |
| SDS | RP | -0.217730275 | 0.95 | -0.474307872 | 0.072939948 | 25,359.23 | 0.129 |
| SDS | BP | -0.27961328 | 0.95 | -0.523830568 | 0.007079283 | 26,647.95 | 0.049 |
| SDS | GH | -0.29198743 | 0.95 | -0.53353897 | -0.006395562 | 26,905.64 | 0.04 |
| SDS | VT | -0.416440539 | 0.95 | -0.627765602 | -0.147942357 | 29,497.37 | 0.003 |
| SDS | SF | -0.449100616 | 0.95 | -0.651508341 | -0.18700439 | 30,177.52 | 0.001 |
| SDS | RE | -0.209155191 | 0.95 | -0.467315015 | 0.081870518 | 25,180.66 | 0.145 |
| SDS | MH | -0.415532987 | 0.95 | -0.62710018 | -0.146868741 | 29,478.47 | 0.003 |
| SDS | VAS | 0.143433258 | 0.95 | -0.148799668 | 0.412625298 | 17,838.00 | 0.32 |
| SDS | PLT | -0.020565267 | 0.95 | -0.304897358 | 0.267132798 | 21,253.27 | 0.887 |
| SDS | FBG | 0.044254626 | 0.95 | -0.244973515 | 0.326249706 | 19,903.40 | 0.76 |
| SDS | DD | 0.235706167 | 0.95 | -0.054066346 | 0.488862624 | 15,916.42 | 0.099 |
| SDS | ESR | 0.061792615 | 0.95 | -0.228371286 | 0.341874298 | 19,538.17 | 0.67 |
| SDS | Hs.CRP | -0.050984895 | 0.95 | -0.332263977 | 0.238622341 | 21,886.76 | 0.725 |
| SDS | RF | -0.153387576 | 0.95 | -0.42103528 | 0.138831684 | 24,019.30 | 0.288 |
| SDS | CCP | -0.260320217 | 0.95 | -0.508566143 | 0.027882804 | 26,246.17 | 0.068 |
| PF | RP | 0.461462419 | 0.95 | 0.202008758 | 0.660392679 | 11,215.05 | <0.001 |
| PF | BP | 0.443936974 | 0.95 | 0.180772925 | 0.647780757 | 11,580.01 | 0.001 |
| PF | GH | 0.332883038 | 0.95 | 0.051679109 | 0.56517797 | 13,892.71 | 0.018 |
| PF | VT | 0.620054432 | 0.95 | 0.405949249 | 0.769636478 | 7,912.37 | <0.001 |
| PF | SF | 0.387478629 | 0.95 | 0.113990294 | 0.606377128 | 12,755.76 | 0.005 |
| PF | RE | 0.378111172 | 0.95 | 0.103143863 | 0.599390765 | 12,950.84 | 0.007 |
| PF | MH | 0.469647909 | 0.95 | 0.212011528 | 0.666245043 | 11,044.58 | <0.001 |
| PF | VAS | -0.184663061 | 0.95 | -0.447162548 | 0.107123186 | 24,670.61 | 0.199 |
| PF | PLT | -0.073701078 | 0.95 | -0.352396004 | 0.217001188 | 22,359.83 | 0.611 |
| PF | FBG | 0.005992632 | 0.95 | -0.28061471 | 0.291618795 | 20,700.20 | 0.967 |
| PF | DD | -0.059431673 | 0.95 | -0.339779916 | 0.230616131 | 22,062.67 | 0.682 |
| PF | ESR | -0.135138089 | 0.95 | -0.405581827 | 0.157061108 | 23,639.25 | 0.349 |
| PF | Hs.CRP | -0.06792338 | 0.95 | -0.347299911 | 0.222527553 | 22,239.50 | 0.639 |
| PF | RF | -0.047812539 | 0.95 | -0.329431958 | 0.241619098 | 21,820.70 | 0.742 |
| PF | CCP | 0.051282628 | 0.95 | -0.238340805 | 0.332529508 | 19,757.04 | 0.724 |
| RP | BP | -0.043980866 | 0.95 | -0.326004585 | 0.24523133 | 21,740.90 | 0.762 |
| RP | GH | -0.027926917 | 0.95 | -0.311563517 | 0.260279061 | 21,406.58 | 0.847 |
| RP | VT | 0.249251348 | 0.95 | -0.039706607 | 0.499737581 | 15,634.34 | 0.081 |
| RP | SF | 0.058545257 | 0.95 | -0.231458161 | 0.338992862 | 19,605.80 | 0.686 |
| RP | RE | 0.647089835 | 0.95 | 0.442981357 | 0.787424828 | 7,349.35 | <0.001 |
| RP | MH | 0.283904247 | 0.95 | -0.002418391 | 0.527204339 | 14,912.69 | 0.046 |
| RP | VAS | -0.089941209 | 0.95 | -0.366632284 | 0.201367065 | 22,698.03 | 0.534 |
| RP | PLT | 0.003831973 | 0.95 | -0.282604068 | 0.289640589 | 20,745.20 | 0.979 |
| RP | FBG | -0.064919263 | 0.95 | -0.344643659 | 0.225393633 | 22,176.94 | 0.654 |
| RP | DD | -0.189641478 | 0.95 | -0.451280553 | 0.102020482 | 24,774.28 | 0.187 |
| RP | ESR | -0.071102953 | 0.95 | -0.350106429 | 0.2194886 | 22,305.72 | 0.624 |
| RP | Hs.CRP | -0.149169981 | 0.95 | -0.417477622 | 0.14306231 | 23,931.47 | 0.301 |
| RP | RF | -0.035759927 | 0.95 | -0.31862598 | 0.252954452 | 21,569.70 | 0.805 |
| RP | CCP | 0.051385472 | 0.95 | -0.238243544 | 0.332621219 | 19,754.90 | 0.723 |
| BP | GH | 0.5398592 | 0.95 | 0.300086774 | 0.715468502 | 9,582.43 | <0.001 |
| BP | VT | 0.629656406 | 0.95 | 0.419021658 | 0.775980945 | 7,712.41 | <0.001 |
| BP | SF | 0.475211514 | 0.95 | 0.218841276 | 0.670209066 | 10,928.72 | <0.001 |
| BP | RE | 0.131789553 | 0.95 | -0.160384482 | 0.40272944 | 18,080.48 | 0.362 |
| BP | MH | 0.423619983 | 0.95 | 0.156457862 | 0.633018746 | 12,003.11 | 0.002 |
| BP | VAS | -0.155324502 | 0.95 | -0.422666377 | 0.136885192 | 24,059.63 | 0.281 |
| BP | PLT | 0.132211575 | 0.95 | -0.159965996 | 0.403089221 | 18,071.69 | 0.36 |
| BP | FBG | 0.203755895 | 0.95 | -0.08746977 | 0.462895331 | 16,581.78 | 0.156 |
| BP | DD | 0.076378923 | 0.95 | -0.214433495 | 0.354752348 | 19,234.41 | 0.598 |
| BP | ESR | 0.129509168 | 0.95 | -0.162643946 | 0.40078392 | 18,127.97 | 0.37 |
| BP | Hs.CRP | 0.097643326 | 0.95 | -0.193900102 | 0.373339049 | 18,791.58 | 0.5 |
| BP | RF | -0.178651852 | 0.95 | -0.442175371 | 0.11326405 | 24,545.43 | 0.214 |
| BP | CCP | 0.197253583 | 0.95 | -0.09418855 | 0.457555599 | 16,717.19 | 0.17 |
| GH | VT | 0.526405527 | 0.95 | 0.282887388 | 0.706169577 | 9,862.61 | <0.001 |
| GH | SF | 0.533355541 | 0.95 | 0.291752884 | 0.710981052 | 9,717.87 | <0.001 |
| GH | RE | 0.077116329 | 0.95 | -0.213725715 | 0.355400601 | 19,219.05 | 0.595 |
| GH | MH | 0.464668069 | 0.95 | 0.205919665 | 0.662687499 | 11,148.29 | <0.001 |
| GH | VAS | 0.010260234 | 0.95 | -0.276678203 | 0.295518859 | 20,611.33 | 0.944 |
| GH | PLT | -0.141457695 | 0.95 | -0.410950752 | 0.150770906 | 23,770.86 | 0.327 |
| GH | FBG | 0.158939747 | 0.95 | -0.133246046 | 0.425706169 | 17,515.08 | 0.27 |
| GH | DD | 0.260864077 | 0.95 | -0.027299765 | 0.508998587 | 15,392.51 | 0.067 |
| GH | ESR | 0.029522335 | 0.95 | -0.258789879 | 0.313004537 | 20,210.20 | 0.839 |
| GH | Hs.CRP | 0.152545213 | 0.95 | -0.139677503 | 0.420325381 | 17,648.25 | 0.29 |
| GH | RF | -0.018910162 | 0.95 | -0.303394772 | 0.268669714 | 21,218.80 | 0.896 |
| GH | CCP | 0.121976364 | 0.95 | -0.170085929 | 0.394339841 | 18,284.84 | 0.399 |
| VT | SF | 0.68144729 | 0.95 | 0.491069864 | 0.809698527 | 6,633.86 | <0.001 |
| VT | RE | 0.4056787 | 0.95 | 0.135251921 | 0.619855028 | 12,376.74 | 0.003 |
| VT | MH | 0.738405171 | 0.95 | 0.573432012 | 0.845827429 | 5,447.71 | <0.001 |
| VT | VAS | -0.064699356 | 0.95 | -0.344449039 | 0.225603238 | 22,172.36 | 0.655 |
| VT | PLT | -0.096486291 | 0.95 | -0.372333375 | 0.195023971 | 22,834.33 | 0.505 |
| VT | FBG | 0.139282853 | 0.95 | -0.152938303 | 0.409105186 | 17,924.44 | 0.335 |
| VT | DD | 0.07045022 | 0.95 | -0.220112926 | 0.349530688 | 19,357.87 | 0.627 |
| VT | ESR | 0.034863172 | 0.95 | -0.253794684 | 0.317819029 | 20,098.97 | 0.81 |
| VT | Hs.CRP | 0.018665836 | 0.95 | -0.268896468 | 0.303172842 | 20,436.28 | 0.898 |
| VT | RF | -0.043569736 | 0.95 | -0.325636395 | 0.245618438 | 21,732.34 | 0.764 |
| VT | CCP | 0.14880254 | 0.95 | -0.143430382 | 0.417167285 | 17,726.19 | 0.302 |
| SF | RE | 0.32860648 | 0.95 | 0.046888927 | 0.561901086 | 13,981.77 | 0.02 |
| SF | MH | 0.655327344 | 0.95 | 0.454404956 | 0.792798758 | 7,177.81 | <0.001 |
| SF | VAS | -0.085300324 | 0.95 | -0.362577213 | 0.205849972 | 22,601.38 | 0.556 |
| SF | PLT | -0.049952826 | 0.95 | -0.331343186 | 0.239597888 | 21,865.27 | 0.73 |
| SF | FBG | 0.251211655 | 0.95 | -0.037618481 | 0.501304929 | 15,593.52 | 0.078 |
| SF | DD | 0.147763671 | 0.95 | -0.144470595 | 0.416289529 | 17,747.82 | 0.306 |
| SF | ESR | 0.13438957 | 0.95 | -0.157804577 | 0.404944672 | 18,026.34 | 0.352 |
| SF | Hs.CRP | 0.11674453 | 0.95 | -0.175235153 | 0.389848351 | 18,393.80 | 0.419 |
| SF | RF | 0.162538259 | 0.95 | -0.129615903 | 0.428725924 | 17,440.14 | 0.259 |
| SF | CCP | 0.318202546 | 0.95 | 0.035289314 | 0.553898449 | 14,198.43 | 0.024 |
| RE | MH | 0.466097233 | 0.95 | 0.207665918 | 0.663709393 | 11,118.53 | <0.001 |
| RE | VAS | 0.14120074 | 0.95 | -0.151027128 | 0.410732815 | 17,884.50 | 0.328 |
| RE | PLT | -0.023575431 | 0.95 | -0.307626508 | 0.264333832 | 21,315.96 | 0.871 |
| RE | FBG | 0.191779023 | 0.95 | -0.099824853 | 0.453045256 | 16,831.20 | 0.182 |
| RE | DD | 0.035282893 | 0.95 | -0.253401472 | 0.318196768 | 20,090.23 | 0.808 |
| RE | ESR | 0.020065956 | 0.95 | -0.267596608 | 0.304444208 | 20,407.13 | 0.89 |
| RE | Hs.CRP | -0.06217365 | 0.95 | -0.342212052 | 0.228008699 | 22,119.77 | 0.668 |
| RE | RF | -0.032499862 | 0.95 | -0.315690424 | 0.256006948 | 21,501.81 | 0.823 |
| RE | CCP | 0.191182297 | 0.95 | -0.10043808 | 0.45255282 | 16,843.63 | 0.184 |
| MH | VAS | 0.111458685 | 0.95 | -0.180421364 | 0.385297261 | 18,503.87 | 0.441 |
| MH | PLT | -0.146650983 | 0.95 | -0.415348846 | 0.145584008 | 23,879.01 | 0.31 |
| MH | FBG | 0.036505327 | 0.95 | -0.252255703 | 0.31929642 | 20,064.78 | 0.801 |
| MH | DD | 0.070232435 | 0.95 | -0.22032118 | 0.349338545 | 19,362.41 | 0.628 |
| MH | ESR | -0.092124306 | 0.95 | -0.368536182 | 0.199254053 | 22,743.49 | 0.525 |
| MH | Hs.CRP | -0.064085716 | 0.95 | -0.343905838 | 0.22618799 | 22,159.59 | 0.658 |
| MH | RF | 0.042418181 | 0.95 | -0.246702218 | 0.324604656 | 19,941.64 | 0.77 |
| MH | CCP | 0.034654591 | 0.95 | -0.253990056 | 0.317631277 | 20,103.32 | 0.811 |
| VAS | PLT | -0.237280029 | 0.95 | -0.490130276 | 0.052403969 | 25,766.36 | 0.097 |
| VAS | FBG | 0.039511292 | 0.95 | -0.249434808 | 0.32199723 | 20,002.18 | 0.785 |
| VAS | DD | 0.124580577 | 0.95 | -0.167516876 | 0.396570697 | 18,230.61 | 0.389 |
| VAS | ESR | -0.1142206 | 0.95 | -0.387676921 | 0.177713532 | 23,203.64 | 0.43 |
| VAS | Hs.CRP | -0.072551019 | 0.95 | -0.351382936 | 0.218102706 | 22,335.88 | 0.617 |
| VAS | RF | -0.005619443 | 0.95 | -0.291277293 | 0.280958488 | 20,942.03 | 0.969 |
| VAS | CCP | -0.083862422 | 0.95 | -0.361318681 | 0.207236452 | 22,571.44 | 0.563 |
| PLT | FBG | 0.338627556 | 0.95 | 0.058133984 | 0.569568163 | 13,773.08 | 0.016 |
| PLT | DD | 0.344890591 | 0.95 | 0.06519832 | 0.574339659 | 13,642.65 | 0.014 |
| PLT | ESR | 0.365938363 | 0.95 | 0.089146188 | 0.590261487 | 13,204.33 | 0.009 |
| PLT | Hs.CRP | 0.397848434 | 0.95 | 0.126073845 | 0.614071841 | 12,539.81 | 0.004 |
| PLT | RF | 0.081644415 | 0.95 | -0.209372847 | 0.359375375 | 19,124.76 | 0.573 |
| PLT | CCP | -0.094164608 | 0.95 | -0.370313454 | 0.197276799 | 22,785.98 | 0.515 |
| FBG | DD | 0.626337172 | 0.95 | 0.414492858 | 0.773791117 | 7,781.53 | <0.001 |
| FBG | ESR | 0.806514666 | 0.95 | 0.676501273 | 0.887773769 | 4,029.33 | <0.001 |
| FBG | Hs.CRP | 0.727417017 | 0.95 | 0.557279016 | 0.838933425 | 5,676.54 | <0.001 |
| FBG | RF | 0.058283694 | 0.95 | -0.231706545 | 0.338760543 | 19,611.24 | 0.688 |
| FBG | CCP | 0.027075119 | 0.95 | -0.261073579 | 0.310793618 | 20,261.16 | 0.852 |
| DD | ESR | 0.535554626 | 0.95 | 0.294566726 | 0.712500015 | 9,672.08 | <0.001 |
| DD | Hs.CRP | 0.639183299 | 0.95 | 0.432078729 | 0.782246675 | 7,514.01 | <0.001 |
| DD | RF | 0.083123138 | 0.95 | -0.207948842 | 0.360671226 | 19,093.96 | 0.566 |
| DD | CCP | -0.179566719 | 0.95 | -0.44293544 | 0.112330887 | 24,564.48 | 0.212 |
| ESR | Hs.CRP | 0.682390307 | 0.95 | 0.492406326 | 0.810304712 | 6,614.22 | <0.001 |
| ESR | RF | 0.040437588 | 0.95 | -0.248564553 | 0.322828566 | 19,982.89 | 0.78 |
| ESR | CCP | 0.033374243 | 0.95 | -0.255188804 | 0.3164783 | 20,129.98 | 0.818 |
| Hs.CRP | RF | 0.237262905 | 0.95 | -0.052422065 | 0.49011649 | 15,884.00 | 0.097 |
| Hs.CRP | CCP | 0.078242928 | 0.95 | -0.212643789 | 0.356390476 | 19,195.59 | 0.589 |
| RF | CCP | 0.21497581 | 0.95 | -0.075813693 | 0.472065169 | 16,348.13 | 0.134 |
